# Nonpeptidic Z360-Analogs Tagged with Trivalent Radiometals as Anti-CCK_2_R Cancer Theranostic Agents: A Preclinical Study

**DOI:** 10.3390/pharmaceutics14030666

**Published:** 2022-03-18

**Authors:** Berthold A. Nock, Panagiotis Kanellopoulos, Oleg G. Chepurny, Maritina Rouchota, George Loudos, George G. Holz, Eric P. Krenning, Theodosia Maina

**Affiliations:** 1 Molecular Radiopharmacy, INRASTES, NCSR “Demokritos”, 15341 Athens, Greece; nock_berthold.a@hotmail.com (B.A.N.); kanelospan@gmail.com (P.K.); 2 Departments of Medicine and Pharmacology, State University of New York (SUNY), Upstate Medical University, Syracuse, NY 13210, USA and Department of Chemistry, Syracuse University, Syracuse, NY 13244, USA; chepurno@upstate.edu (O.G.C.); holzg@upstate.edu (G.G.H.); 3 BIOEMTECH, Lefkippos Attica Technology Park NCSR “Demokritos”, 15310 Athens, Greece; mrouchota@bioemtech.com (M.R.); george@bioemtech.com (G.L.); 4 Cyclotron Rotterdam BV, Erasmus MC, 3015 CE Rotterdam, The Netherlands; erickrenning@gmail.com

**Keywords:** tumor theranostics, CCK_2_R-radioligand, Z360, nonpeptidic radioligand, SPECT, PET, radionuclide therapy, [^111^In]In/[^67/68^Ga]Ga/[^177^Lu]Lu-radioligand

## Abstract

(1) Background: Theranostic approaches in the management of cholecystokinin subtype 2 receptor (CCK_2_R)-positive tumors include radiolabeled gastrin and CCK motifs. Moving toward antagonist-based CCK_2_R-radioligands instead, we herein present three analogs of the nonpeptidic CCK_2_R-antagonist Z360, GAS1/2/3. Each was conjugated to a different chelator (DOTA, NODAGA or DOTAGA) for labeling with medically relevant trivalent radiometals (e.g., Ga-68, In-111, Lu-177) for potential use as anti-CCK_2_R cancer agents; (2) Methods: The in vitro properties of the thee analogs were compared in stably transfected HEK293-CCK_2_R cells. Biodistribution profiles were compared in SCID mice bearing twin HEK293-CCK_2_R and wtHEK293 tumors; (3) Results: The GAS1/2/3 analogs displayed high CCK_2_R-affinity (lower nM-range). The radioligands were fairly stable in vivo and selectively targeted the HEK293-CCK_2_R, but not the CCK_2_R-negative wtHEK293 tumors in mice. Their overall pharmacokinetic profile was found strongly dependent on the radiometal-chelate. Results could be visualized by SPECT/CT for the [^111^In]In-analogs; (4) Conclusions: The present study highlighted the high impact of the radiometal-chelate on the end-pharmacokinetics of a new series of Z360-based radioligands, revealing candidates with promising properties for clinical translation. It also provided the impetus for the development of a new class of nonpeptidic radioligands for CCK_2_R-targeted theranostics of human cancer.

## 1. Introduction

The cholecystokinin subtype 2 receptor (CCK_2_R) represents a clinically relevant biomolecular target for nuclear medicine applications owing to its overexpression in a variety of human tumors, such as medullary thyroid carcinoma (MTC), small cell lung cancer (SCLC), astrocytomas, stromal ovarian cancers or gastrointestinal stromal tumors (GIST) [1,2,3,4,5,6]. In view of that, a good number of peptide radioligands were previously developed with the aim to deliver diagnostic or therapeutic radionuclides specifically on CCK_2_R-positive cancer lesions [7,8]. Thus far, the native peptides cholecystokinin (e.g., sulfated CCK-8, H-Asp-Tyr(SO_3_H)-Met-Gly-Trp-Met-Asp-Phe-NH_2_) and gastrin (gastrin-17, pGlu-Gly-Pro-Trp-Leu-Glu-Glu-Glu-Glu-Glu-Ala-Tyr-Gly-Trp-Met-Asp-Phe-NH_2_) have served as motifs in anti-CCK_2_R radioligand design. The coupling of suitable chelators has enabled labeling with radiometals clinically applied for diagnosis and therapy–“theranostics”. Following this concept, diagnostic imaging with single-photon emission tomography (SPECT: In-111) or positron emission tomography (PET: Ga-68) serves to select patients eligible for radionuclide therapy with particle emitters (e.g., Lu-177, Y-90 or Ac-225). Targeted radiotherapy aims at the eradication of tumor cells only, sparing healthy tissues devoid of CCK_2_R-expression. Thereafter, imaging is applied again to monitor therapeutic responses and disease progression in a patient-tailored protocol.

A major challenge in the development of clinically useful CCK_2_R-radioligands is the fast in vivo degradation of truncated *des*-(Glu)_5_ gastrin and minigastrin (MG: gastrin(4–17)) analogs [9,10,11,12,13,14]. Notably, the presence of the (Glu)_5_-chain, although shown to favor metabolic stability and tumor targeting of related radioligands, led to prolonged kidney retention. In a smart, recent approach, the administration of key protease inhibitors successfully stabilized *des*-(Glu)_5_-gastrin radioligands in vivo, significantly enhancing CCK_2_R-positive tumor uptake in mice and MTC patients [15,16]. In another approach, innovative structural interventions on the peptide chain led to metabolic stability improvements without compromising other important properties of resulting radioligands [17,18,19,20,21]. The clinical value of the selected new analogs is currently being assessed through pilot studies in MTC patients [22,23,24]. Thus far, all tested radioligands have been peptide-based, displaying agonistic properties at the CCK_2_R. Consequently, they are prone to elicit adverse effects after binding and activation of the CCK_2_R when injected to patients. These effects are well described during the provocative pentagastrin (Boc-ßAla-Trp-Met-Asp-Phe-NH_2_) test used in the early diagnosis and follow up of MTC [25]. Another point of concern is the tendency of radiolabeled gastrin analogs to accumulate in the gastric mucosa, a tissue of high physiological CCK_2_R-expression levels, a feature harboring dosimetric restrictions, especially during radiotherapy [17,26].

It is reasonable to assume that such concerns can be efficiently addressed by the use of radiolabeled CCK_2_R-antagonists, based on the recent positive experience from the fields of somatostatin and bombesin radioligands. It should be noted that in the case of CCK_2_R, the most known antagonists considered as motifs in radioligand design are non-peptidic small organic molecules [27]. Benzodiazepines represent a major class thereof, with Z360 (3-[[1-cyclohexyl-5-(3,3-dimethyl-2-oxobutyl)-4-oxo-2,3-dihydro-1,5-benzo-diazepin-3-yl]-carbamoylamino-benzoic acid) in particular having recently attracted our attention. Z360 was first reported as a CCK_2_R-antagonist, shown to inhibit the meal- or pentagastrin-induced acid secretion in the gastric mucosa in dogs after systemic administration [28,29]. Next, Z360 was shown to play a role in several gastrointestinal pathological processes, including cancer, and was eventually proposed as a drug against pancreatic cancer [30,31,32,33,34].

**Figure 1:**
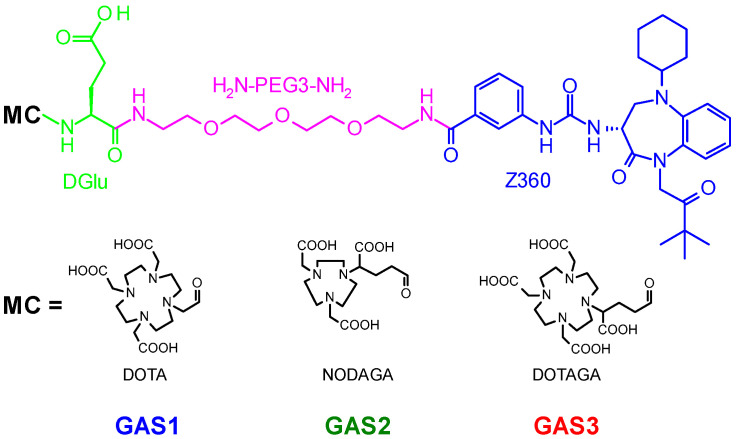
Chemical structure of GAS1, GAS2 and GAS3.

The feasibility of attaching functional groups, such as cytotoxic drugs, dyes or metal-chelates, at the free carboxylic acid of Z360, without compromising the affinity of resulting bioconjugates for the CCK_2_R, was recently established [35,36,37]. We previously reported on [^99m^Tc]Tc-DGA1 (DGA1: Z360-PEG_3_-DGlu-DGlu-DGlu-DGu-Lys(N_4_)−OH; N_4_: 6-carboxy-1,4,8,11-tetraazaundecane), our first Z360 [^99m^Tc]Tc-based radioligand, suitable for imaging CCK_2_R-positive tumors with SPECT and SPECT/CT [38]. Aiming to broaden the applicability of Z360 in PET imaging and radionuclide therapy, we now present three new analogs, GAS1/2/3 (Figure 1), amenable for labeling with theranostic trivalent radiometals. For this purpose, the macrocyclic chelators, DOTA (1,4,7,10-tetraazacyclododecane-1,4,7,10-tetraacetic acid), NODAGA (1,4,7-triazacyclononane,1-glutaric acid-4,7-acetic acid) or DOTAGA (1,4,7,10-tetraazacyclododececane,1-(glutaric acid)-4,7,10-triacetic acid) were coupled at the pendant carboxylate group of Z360 via the common H-DGlu-HN(PEG3)NH_2_ (α,ω-diamino tri-(ethylene glycol)) linker. This arrangement has allowed for the direct comparison of biological responses across analogs after labeling them with representative radiometals used in cancer theranostics, specifically for PET (Ga-67 as a surrogate of Ga-68), SPECT (In-111) and radionuclide therapy (Lu-177). Accordingly, pharmacological features of resulting analogs could be directly compared in CCK_2_R-positive cell preparations and their in vivo profiles in mice models. In this way, radioligands were screened to select candidates for further clinical evaluation. Moreover, conclusions on the strengths and limitations of using Z360-based radioligands, and possibly CCK_2_R-antagonists in general, in cancer theranostics could be drawn.

## 2. Materials and Methods

### 2.1. Chemicals and Ligands

Z360 was purchased from MedKoo Biosciences, Inc. (Morrisville, NC/USA). The three Z360-conjugates (Figure 1): (i) GAS1, DOTA-DGlu-HN(PEG3)NH-Z360, (ii) GAS2, NODAGA-DGlu-HN(PEG3)NH-Z360 and (iii) GAS3, DOTAGA-DGlu-HN(PEG3)NH-Z360, as well as DG2 were synthesized by PiChem Forschungs- und Entwickungs GmbH (Raaba-Grambach, Austria); analytical data, comprising MALDI-TOF mass spectroscopy results and results from HPLC analysis in two distinct HPLC systems, is compiled in Table S1 (Supplementary File). The human [Leu^15^]gastrin-17 (pGlu-Gly-Pro-Trp-Leu-(Glu)_5_-Ala-Tyr-Gly-Trp-Leu-Asp-Phe-NH_2_) was purchased from Bachem (Bubendorf, Switzerland). For metal incorporation by the GAS-analogs, the nitrate salts [^nat^Ga]Ga(NO_3_)_3_, [^nat^In]In(NO_3_)_3_ and [^nat^Lu]Lu(NO_3_)_3_ were obtained from Sigma-Aldrich. Gelofusine was obtained from B. Braun Melsungen AG (Melsungen, Germany).

### 2.2. Labeling of GAS-Compounds with Ga-67, In-111 and Lu-177

All three GAS analogs were labeled with Ga-67 (serving as a surrogate of the PET radionuclide Ga-68) and In-111, but only GAS1 and GAS3 were labeled with Lu-177. Lyophilized GAS1/2/3 were dissolved in HPLC-grade H_2_O (2 mg/mL), and 50 μL aliquots thereof were stored in Eppendorf Protein LoBind tubes at –20 °C. For Ga-67 labeling, [^67^Ga]GaCl_3_ (4–5.5 GBq/mL in dilute HC1 solution) was provided by IDB Holland B.V. (Baarle-Nassau, The Netherlands). For labeling with In-111, [^111^In]InCl_3_ (400–600 MBq/mL in 0.05 mM HCl) was purchased from Curium Pharma (Petten, The Netherlands). For labeling with Lu-177 [^177^Lu]LuCl_3_ (3.7 GBq/mL in 0.04 M HCl, RLu-3, A_s_ > 370 GBq/mg Lu) were obtained from POLATOM (Otwok, Poland). Labeling protocols for each radionuclide are presented in detail in the Supplementary File.

For the preparation of [^125^I][I-Tyr^12^,Leu^15^]gastrin-17, [^125^I]NaI in a 0.1 M NaOH solution (Perkin Elmer) was employed, as previously described [38]. Aliquots of the radioligand stock solution in 0.1% BSA-PBS buffer were kept at −20 °C and were used in competition binding experiments (molecular activity of 74 GBq/μmol). The preparation of [^nat^In]In/[^nat^Ga]Ga-GAS1/2/3 and [^nat^Lu]Lu-GAS1/3 is detailed in the Supplementary File.

### 2.3. Quality Control of Radiolabeled GAS1/2/3

Reversed-phase high-performance liquid chromatography (RP-HPLC) was performed on a Waters Chromatograph based on a 600 E multi-solvent delivery system coupled to a Waters 2998 photodiode array detector (Waters, Vienna, Austria) and a Gabi gamma-detector (Raytest, RSM Analytische Instrumente GmbH, Straubenhardt, Germany). The processing of data and chromatography were controlled by Empower Software (Waters, Milford, MA, USA). For quality control, aliquots of the radiolabeling solution were loaded on a Symmetry Shield RP18 cartridge column (5 μm, 3.9 mm × 150 mm, Waters, Eschborn, Germany), eluted with the following linear gradient: 100%A/0% B to 70%A/30% B in 5 min and then 70%A/30% B to 55%A/45% B in 60 min, whereby A = 0.1% TFA in H_2_O (*v*/*v*) and B = MeCN (system 1). The radiochemical labeling yields exceeded 98%, and the radiochemical purity was >99%; therefore, radioligands were used without further purification in all subsequent experiments. Samples of [^111^In]In/[^67^Ga]Ga-GAS1/2/3 and [^177^Lu]Lu-GAS1/3 were tested before and after the end of all biological experiments.

Handling of solutions containing beta-/gamma-emitting radionuclides was conducted by authorized personnel in compliance with European radiation safety guidelines. Licensed facilities were supervised by the Greek Atomic Energy Commission (GAEC, license #A/435/17092/2019 and #A/435/15767/2019).

### 2.4. Cell Culture

For most biological experiments, HEK293 cells transfected to stably express the human CCK_2i4sv_R splice variant were used, kindly provided by Dr. P. Laverman (Radboud University Nijmegen Medical Center, Nijmegen, The Netherlands) and Prof. M. R. Hellmich (University of Texas Medical Branch, Galveston, TX, USA) [39,40,41], while non-transfected wtHEK293 cells served as negative controls. Cells were cultured in DMEM with GlutaMAX-I supplemented with 10% (*v*/*v*) fetal bovine serum (FBS), 100 U/mL penicillin and 100 μg/mL streptomycin and kept in a controlled humidified air containing 5% CO_2_ at 37 °C; the medium for transfected cells was additionally supplemented with 400 μg/mL G418. All culture media were purchased from Gibco BRL, Life Technologies (Grand Island, NY, USA) and supplements were supplied by Biochrom KG Seromed (Berlin, Germany). For Ca^2+^-mobilization assays, HEK293 cell clones (generated by O.G. Chepurny in the G.G. Holz laboratory) were transfected to stably co-express either both the human CCK_2_R and the Ca^2+^ biosensor YC3.60 (clone #21) or only YC3.60 (clone #5, negative controls devoid of CCK_2_R expression) (Supplementary File). Ca^2+^ mobilization assays were conducted by FRET-based detection of Ca^2+^ in HEK293-hCCK_2_R/YC3.60 and HEK293-YC3.60 cells (negative controls) for GAS1/2/3 as well as for DG2, [Leu^15^]gastrin-17 and Z360 (controls), as outlined in the Supplementary File.

### 2.5. Competition Binding Assays in HEK293-CCK_2i4sv_R Cell Membranes

Competition binding assays for the metal-free GAS1/2/3 and the corresponding metal-tagged [^nat^Ga]Ga/[^nat^In]In/[^nat^Lu]Lu-GAS1/3 and [^nat^Ga]Ga/[^nat^In]In-GAS2 analogs were conducted against the [^125^I][I-Tyr^12^,Leu^15^]gastrin-17 radioligand in membrane homogenates harvested from HEK293-CCK_2i4sv_R cells, as previously described [38,42]. Briefly, each of the above analogs, or unmodified Z360 serving as control, was incubated in triplicates of increasing concentrations (10^−13^–10^−6^ M) with [^125^I][I-Tyr^12^,Leu^15^]gastrin-17 (50 pM, ≈40,000 cpm) and membrane homogenate in binding buffer (300 µL, pH 7.4, 50 mM HEPES, 1% BSA, 5.5 mM MgCl_2_, 35 µM bacitracin) for 1 h at 22 °C. Incubation was interrupted by rapid filtration through glass fiber filters (Whatman GF/B, presoaked in binding buffer for at least 1 h) on a Brandel Cell Harvester (Adi Hassel Ingenieur Büro, Munich, Germany) and subsequent rinsing with ice-cold washing buffer (10 mM HEPES pH 7.4, 150 mM NaCl). The radioactivity content of individual filters was measured in a gamma counter (automated multi-sample well-type instrument with a NaI(Tl) 3′ crystal, Canberra Packard Cobra^TM^ Quantum U5003/1, Auto-Gamma^®^ counting system). The 50% inhibitory concentration (IC_50_) was determined by nonlinear regression analysis according to a one-site model using PRISM 6 (Graph Pad Software, San Diego, CA, USA). Values are expressed as mean ± standard deviation (SD) of three independent experiments performed in triplicate.

### 2.6. Uptake of Radiolabeled GAS1/2/3 in HEK293-CCK_2i4sv_R Cells

A day before the experiment, HEK293-CCK_2i4sv_R cells were seeded in six-well plates. The next day, cells were rinsed with ice-cold internalization medium (IM: DMEM Glutamax-I, supplemented by 1% (*v*/*v*) FBS) and then the fresh medium was added (1.2 mL) at 37 °C. A further portion of IM (150 µL) was added in the upper well-row, and DG2 solution in IM (150 µL) was added in the lower row (to a final concentration of 1 μM; non-specific series). Each of the test radioligands, [^67^Ga]Ga/[^111^In]In/[^177^Lu]Lu-GAS1/3 and [^67^Ga]Ga/[^111^In]In-GAS2, was finally added (250 fmol total conjugates in 150 μL 0.5% BSA-PBS) in all the wells and the plates were incubated at for 1 h at 37 °C in an Incubator-Orbital Shaker unit (MPM Instr. SrI, Bernareggio, MI, Italy); [^111^In]In-GAS1/2/3 were incubated for 15 min, 30 min, 1 h and 2 h. At the predetermined time points, the plates were placed on ice, the medium was collected, and the plates were rinsed with 0.5% BSA-PBS (1 mL). Cells were subsequently treated with an acid-wash solution (2 × 600 µL; 50 mM glycine buffer pH 2.8, 0.1 M NaCl) and the fractions collected (membrane-bound). After briefly washing with 0.5% BSA-PBS (1 mL), the cells were lysed with 1 N NaOH (2 × 600 µL), and the fractions were collected (internalized). The radioactivity content of collected fractions was counted in the gamma counter, and the percentage of internalized and membrane-bound fractions per time point were calculated with Microsoft Excel. Specific internalized and membrane-bound counts were determined by subtracting the respective non-specific (in the presence of 1 μM DG2) from the respective total counts. Results represent specific internalized/membrane bound ± SD of total added radioactivity per well from three experiments performed in triplicate.

### 2.7. Radioligand Stability in Mice

The formation of radiometabolites which can be detected in the blood was tested in blood samples collected 5 min post-injection (pi) of each of the radioligands [^67^Ga]Ga/[^111^In]In/[^177^Lu]Lu-GAS1/3 and [^67^Ga]Ga/[^111^In]In-GAS2. For this study, in-house male Swiss albino mice (body weight: 30 ± 5 g, provided by the NCSR “Demokritos” Animal House) were used in groups of three. The animals received, through the tail vein, a 100-μL bolus containing each radioligand tested (2.5–3 nmol of the total conjugate in vehicle: saline/EtOH 9/1 *v*/*v*, corresponding to: up to 11 MBq for In-111, up to 13 MBq for Ga-67 and up to 74 MBq for Lu-177). Mice were euthanized, and blood was collected directly from the heart using a prechilled penicillin syringe and transferred in a prechilled EDTA-containing Eppendorf Protein LoBind^®^ tube on ice (40 µL, 50 mM Na_2_EDTA solution). After centrifugation (10 min, 2000× *g*/4 °C, in a Hettich, Universal 320R, centrifuge), the blood plasma was collected, mixed with chilled MeCN in a 1/1 *v*/*v* ratio and centrifuged again (10 min, 15,000× *g*/4 °C). Supernatants were collected and concentrated to a small volume (≈50–100 μL) under a gentle N_2_-flux at 40 °C. They were then diluted with physiological saline (≈400 μL) and filtered through a Millex GV filter (0.22 μm, 13 mm Ø, Millipore, Milford, CT, USA). Aliquots of the filtrate were analyzed by radio-RP-HPLC using a Waters Symmetry Shield RP18 cartridge column (5 μm, 3.9 mm × 20 mm) eluted at a flow rate of 1.0 mL/min with the following gradient: 100% A/0% B to 55% A/45% B in 45 min; A = 0.1% aqueous TFA (*v*/*v*) and B = MeCN (system 2). The elution time (*t*_R_) of each parent radioligand was determined by coinjection of blood samples with an aliquot of the labeling solution on the HPLC column. Results were calculated as the average percentage of intact radioligand ± SD derived from three independent experiments.

Following the same injection protocol, radioligands were administered in additional groups of mice, but this time animals were euthanized 30 min pi. The urine was immediately collected from the bladder using a prechilled syringe and transferred as before in a prechilled EDTA-containing Eppendorf Protein LoBind^®^ centrifuge tube on ice (40 µL, 50 mM Na_2_EDTA solution) and 0.1 mL of physiological saline. Urine proteins were precipitated by adding chilled MeCN in a 1/1 *v*/*v* ratio followed by a 10 min centrifugation step (15,000× *g*/4 °C). The supernatant was then collected and treated following the blood sample workup procedure. Aliquots of the Millex-GV filtrate (containing >90% of total urine activity) were analyzed by radio-HPLC, adopting the aforementioned conditions. Results were calculated as the average percentage of intact radioligand ± SD derived from three independent experiments.

### 2.8. Biodistribution in Mice Bearing Twin HEK293-CCK_2i4sv_R and wtHEK293 Xenografts

Inocula containing a suspension of freshly harvested HEK293-CCK_2i4sv_R/wtHEK293 cells (150 μL, 1.8 × 10^7^/1.4 × 10^7^ cells, respectively, in normal saline) were subcutaneously (sc) injected in the right and left flanks of male SCID mice (24.5 ± 2.3 g body weight, six weeks of age on arrival day, NCSR “Demokritos” Animal House Facility). Well-palpable tumors (0.42 ± 0.37/0.25 ± 0.32 mg, respectively) were grown at the inoculation site 3–4 weeks later, and biodistribution was conducted. On the day of biodistribution, animals were divided into groups of four and received the radioligand through the tail vein as a bolus (100 μL, containing each of: i. 78–91 kBq [^67^Ga]GAS1/2/3 corresponding to 7–17 pmol GAS1/2/3; ii. 74–92 kBq [^111^In]GAS1/2/3 corresponding to 20–25 pmol GAS1/2/3; iii. 730 kBq [^177^Lu]Lu-GAS1/3 corresponding to 40 pmol GAS1/3, in-vehicle: saline/EtOH 9/1 *v*/*v*). Animals were euthanized at predetermined time intervals depending upon the radionuclide applied (for [^67^Ga]Ga-GAS1/2/3: 1, 4 and 24 h pi; for [^111^In]In-GAS1/2/3: 4 and 24 h pi–two extra groups of mice receiving gelofusine together with [^111^In]In-GAS1/2 at 4 and 24 h pi were included; and for [^177^Lu]Lu-GAS1/3: 4, 24, 48, 72 and 96 h pi). Mice were dissected, and blood samples, organs of interest and tumors were rapidly collected, weighted and counted in the gamma counter. Biodistribution data was calculated as the percentage of the administered dose per gram tissue (%IA/g) with the aid of suitable standards of the administered dose and the Microsoft Excel program. Results are presented as mean % IA/g values ± SD, *n* = 4 per time point, as determined by the PRISM^TM^ 6.0 GraphPad software (San Diego, CA, USA).

### 2.9. Statistical Analysis

The statistical evaluation of results was carried out by applying a two-way ANOVA with multiple comparisons and Tukey’s post hoc analysis (PRISM^TM^ 6.0 GraphPad Software, San Diego, CA/USA). *p*-values of <0.05 were considered to be statistically significant.

### 2.10. SPECT/CT of HEK293-CCK_2i4sv_R and wtHEK293 Xenografts with [^111^In]In-GAS1/2/3

For SPECT/CT imaging, six mice bearing twin HEK293-CCK_2i4sv_R and wtHEK293 tumors were injected in the tail vein with a bolus containing [^111^In]In-GAS1/2/3 (100 μL, 9–12 MBq associated with 3 nmol total injected analog in-vehicle: saline/EtOH 9/1 *v*/*v*). Mice were euthanized at 4 h and 24 h pi. Tomographic SPECT/CT imaging was performed with the y-CUBE/x-CUBE systems (Molecubes, Belgium). The SPECT system is based on monolithic NaI detectors attached to SiPMs, with a 0.6 mm intrinsic resolution. The CT system is based on a structured CMOS detector of CsI with pixels of 75 µm and operates between 35–80 kVp, 10–500 µA tube current, with a 33 µm fixed focal spot size. SPECT scans were acquired 4 h pi and 24 h pi, with a 40–50 min duration protocol based on the injected activity, and each SPECT scan was succeeded by a CT scan, following General-Purpose protocol under 50 kVp, for co-registration purposes. SPECT images were reconstructed using the MLEM reconstruction method with a 250 µm voxel size and 500 iterations. CT images were reconstructed using the ISRA reconstruction method with a 100 µm voxel size.

Images were exported and post-processed on VivoQuant software, version 4.0 (Invicro, Boston). A smoothing median filter (0.6 mm, spherical window) was applied to the images, and the bladder was removed for consistency purposes. Normalization of images was performed (i.e., all images having the same color scale range values) to achieve a direct visual comparison between the different groups.

All experiments involving mice were conducted in compliance with European and national regulations in licensed facilities (EL 25 BIO exp021). The study protocols were approved by the Department of Agriculture and Veterinary Service of the Prefecture of Athens (#1609, 24-04-2019 for the stability studies and #1610, 24-04-2019 for the biodistribution and imaging studies).

## 3. Results

### 3.1. Ligands and Radioligands

Analytical data for the Z360 analogs GAS1/2/3, carrying the three different chelators DOTA, NODAGA and DOTAGA via a DGlu-HN(PEG3)NH-linker (Figure 1), comprising results from HPLC analyses in two separate systems and MALDI-TOF mass spectrometry data, are summarized in Table S1 (Supplementary File) and were found to be consistent with the formation of the desired products in high purity (≥95%).

The three bioconjugates, GAS1/2/3, were labeled with Ga-67 (as a Ga-68 surrogate), In-111 and Lu-177, following previously described protocols after a slight modification to avoid by-products caused by the degradation of Z360 at elevated temperatures. Under these precautions, the final radiolabeled products were obtained in >98% purity, as verified by the respective radio-analytical HPLC (Supplementary File).

### 3.2. Receptor Affinity Determination and Functional Studies of Z360-Analogs

The receptor binding affinities of the metal-free GAS1/2/3 and the corresponding metal-tagged [^nat^Ga]Ga/[^nat^In]In/[^nat^Lu]Lu-GAS1/3 and [^nat^Ga]Ga/[^nat^In]In-GAS2 analogs were determined via competition binding assays in HEK293-CCK_2i4sv_R cell membrane homogenates using [^125^I][I-Tyr^12^,Leu^15^]gastrin-17 as the radioligand and unmodified Z360 as control. All tested analogs displaced [^125^I][I-Tyr^12^,Leu^15^]gastrin-17 from HEK293-CCK_2i4sv_R binding sites on the membranes in a mono-phasic and concentration-dependent way.

As summarized in Table 1, the coupling of the metal-chelator led to a drop in the receptor affinities of GAS1/2/3 compared to the unmodified Ζ360; this drop was found to be statistically significant only in the case of the DOTA-modified analog GAS1 (IC_50_ 5.9 ± 1.8 nM vs. IC_50_ 1.2 ± 0.5 nM of Z360; *p* < 0.05). The binding of non-radioactive In, Ga or Lu affected the receptor affinity of the resulting metal-tagged species in different ways. Thus, no statistically significant differences could be observed in the receptor affinities between DOTAGA-modified GAS3 and its [^nat^Ga]Ga/[^nat^In]In/[^nat^Lu]Lu-metal-tagged species. In contrast, in GAS1 (IC_50_ 5.9 ± 1.8 nM), the incorporation of Ga (IC_50_ 13.8 ± 2.7 nM; *p* < 0.0001) or Lu (IC_50_ 12.4 ± 0.2 nM; *p* < 0.0001) led to a clear decline of receptor affinity. Notably, the most drastic drop of receptor affinity was displayed after the incorporation of In by the NODAGA-modified analog GAS2 (53.2 ± 2.7 nM vs. IC_50_ 4.1 ± 1.1 nM of GAS2; *p* < 0.0001). In fact, [^nat^In]In-GAS2 showed, by far, the lowest affinity for the CCK_2i4sv_R amongst this series of Z360 analogs (*p* < 0.0001).

Results from Ca^2+^ mobilization assays of Z360, [Leu^15^]gastrin-17 and DG2 (reference compounds) and GAS1/2/3 are included in Figures S1 and S2, respectively (Supplementary File). Interestingly, in the HEK293-hCCK_2_R/YC3.60 cells, GAS1/2/3 and Z360 displayed agonistic properties only above the 100 nM concentration threshold, while the two reference agonists, [Leu^15^]gastrin-17 and DG2, activated the CCK_2_R at 100 pM and 10 pm, respectively. None of the compounds triggered a response in the HEK293-YC3.60 cells, which were devoid of CCK_2_R expression, thereby confirming the receptor-specificity of the assay.

### 3.3. Uptake of GAS1/2/3-Radioligands in HEK293-CCK_2i4sv_R Cells

**Figure 2:**
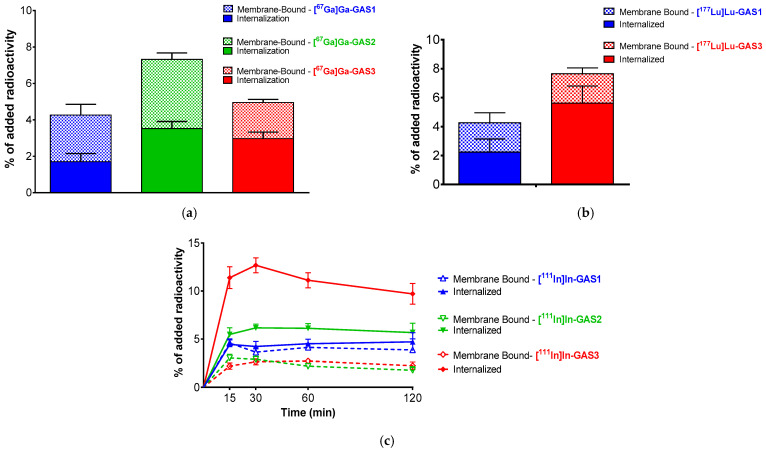
Radioligand uptake-internalization in HEK293-CCK_2i4sv_R cells. (**a**) [^67^Ga]Ga-GAS1/2/3 during 1 h incubation at 37 °C; (**b**) [^177^Lu]Lu-GAS1/3 during 1 h incubation at 37 °C; (**c**) [^111^In]In-GAS1/2/3 during 5, 15, 30 min, 1 and 2 h incubation at 37 °C. Solid bars/lines correspond to internalized fractions and chequered bars/lines to membrane-bound fractions. Results represent the average of 3 independent experiments performed in triplicate.

Comparative radioligand uptake and internalization results in HEK293-CCK_2i4sv_R cells during 1 h incubation at 37 °C are summarized for [^67^Ga]Ga-GAS1/2/3 and [^177^Lu]Lu-GAS1/3 in Figure 2a,b, respectively; the time-dependent uptake and internalization of [^111^In]In-GAS1/2/3 for the 5, 15, 30 min, 1 and 2 h incubation in the same cells are included in Figure 2c.

All analogs displayed receptor-mediated uptake by HEK293-CCK_2i4sv_R cells with a considerable fraction of cell-associated activity found within the cells, ranging between 1.79 ± 0.45% of total added activity for [^67^Ga]Ga-GAS1 to 11.12 ± 0.79% for [^111^In]In-GAS3. The overall cell uptake of the radioligands was found to differ across attached chelators and radiometals. For example, within the sub-group of [^67^Ga]Ga-radioligands, the cell uptake of [^67^Ga]Ga-GAS2 (7.32 ± 0.57%) was found significantly higher to that of [^67^Ga]Ga-GAS1 (4.26 ± 0.99%; *p* < 0.0001) or [^67^Ga]Ga-GAS3 (4.95 ± 0.51%; *p* < 0.0001). On the other hand, (radio)metal incorporation was found to affect cell uptake as well. For example, in the case of GAS3 analogs, cell uptake was found to increase from [^67^Ga]Ga-GAS3 (4.95 ± 0.51%) to [^177^Lu]Lu-GAS3 (7.65 ± 1.31%; *p* < 0.0001) and [^111^In]In-GAS3 (13.81 ± 0.89%; *p* < 0.0001), reaching the maximum value within the whole set of eight radioligands tested herein.

### 3.4. Radioligand Stability in Mice

**Figure 3:**
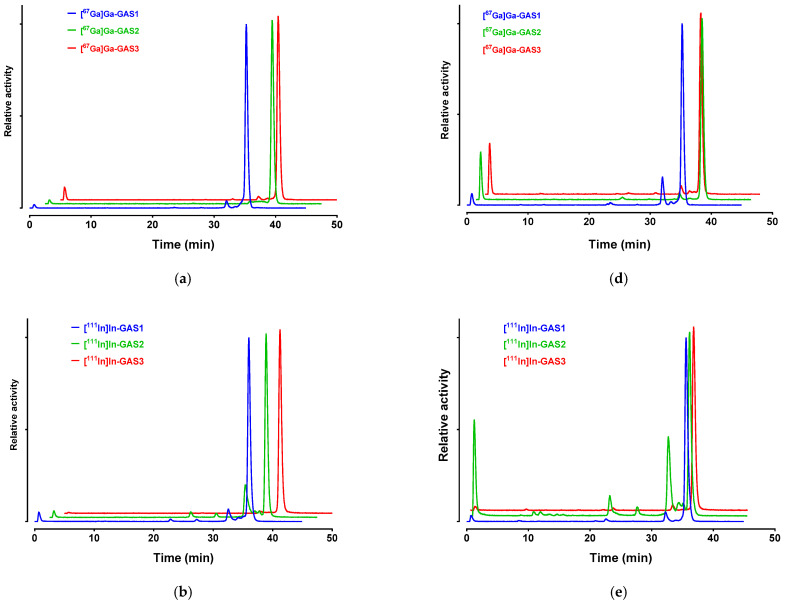

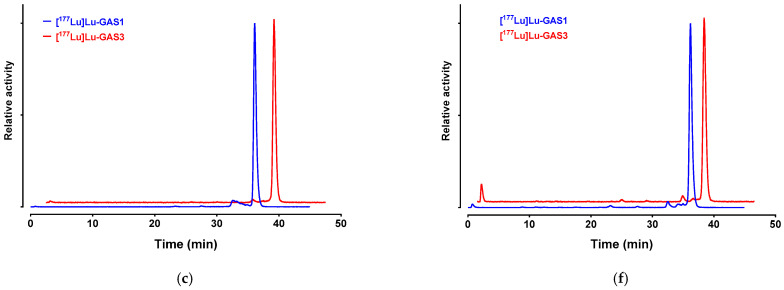
Representative radiochromatograms (system 2) revealing radioligand degradation in mice blood collected 5 min pi for (**a**) [^67^Ga]Ga-GAS1/2/3, (**b**) [^111^In]In-GAS1/2/3 and (**c**) [^177^Lu]Lu-GAS1/3 and in urine collected 30 min pi for (**d**) [^67^Ga]Ga-GAS1/2/3, (**e**) [^111^In]In-GAS1/2/3 and (**f**) [^177^Lu]Lu-GAS1/3.

The in vivo degradation of [^67^Ga]Ga/[^111^In]In/[^177^Lu]Lu-GAS1/3 and [^67^Ga]Ga/[^111^In]In-GAS2 was studied via the radiometric HPLC analysis of blood samples collected 5 min pi and urine samples collected 30 min pi in healthy mice. Representative radiochromatograms of blood and urine analysis are presented in Figure 3a–f, respectively, corresponding to [^67^Ga]Ga/[^111^In]In/[^177^Lu]Lu-radioligands. Numerical values are listed in Table 2.

All radioligands displayed high stability in circulation (>80%) at 5 min pi and were excreted in the urine of mice without any further decomposition at 30 min pi. The only radioligand showing significantly lower stability in the blood (73.1 ± 1.8% intact at 5 min pi) and the urine (44.1 ± 2.6% intact) turned out to be [^111^In]In-GAS2, modified with the NODAGA chelator. In view of these findings, the action of peptidase inhibitors known to prolong the stability of peptide-based radioligands (e.g., the neprilysin inhibitors phosphoramidon, thiorphan or sacubitrilat and/or the angiotensin-converting enzyme inhibitor lisinopril) on the stability and biodistribution of the GAS1/2/3 radioligands were not investigated in the present study [9,12,15,43].

### 3.5. Biodistribution in Mice Bearing Twin HEK293-CCK_2i4sv_R and wtHEK293 Xenografts

**Figure 4:**
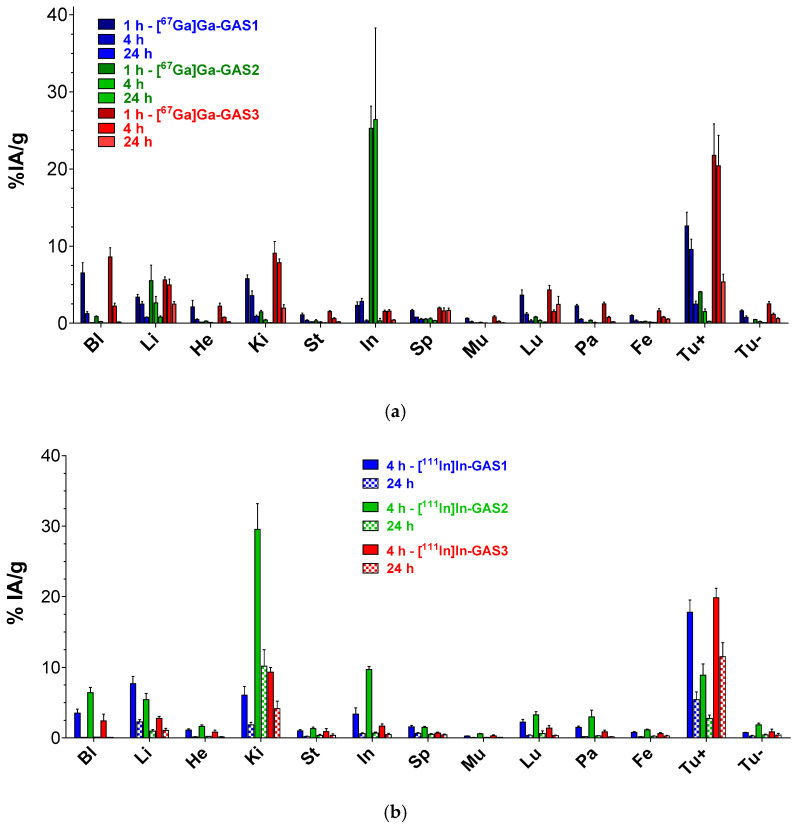

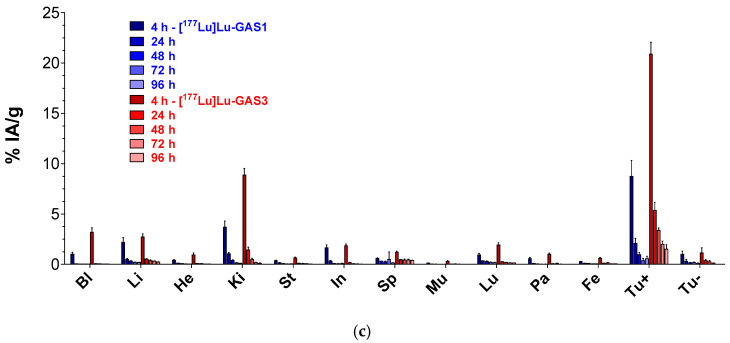
Comparative biodistribution data in SCID mice bearing twin HEK293-CCK_2i4sv_R and wtHEK293 tumors in their flanks expressed as % IA/g tissue (mean ± SD, *n* = 4) for (**a**) [^67^Ga]Ga-GAS1/2/3, (**b**) [^111^In]In-GAS1/2/3 and (**c**) [^177^Lu]Lu-GAS1/3; Bl: blood, Li: liver, He: heart, Ki: kidneys, St: stomach, In: intestines, Sp: spleen, Mu: muscle, Lu: lungs, Pa: pancreas, Fe: femur, Tu+/−: HEK293-CCK_2i4sv_R/wtHEK293 tumor.

Biodistribution results for GAS1/2/3 labeled with Ga-67, In-111 and GAS1/3 labeled with Lu-177 in SCID mice bearing double HEK293-CCK_2i4sv_R and wtHEK293 subcutaneous tumors are summarized in Figure 4a–c, respectively. Data are expressed as %IA/g and represents average values ± SD, *n* = 4 per animal group. Results in numerical values are separately displayed in Tables S2–S9 in the Supplementary File. In all cases, a notable uptake of the radioligands is evident only in the HEK293-CCK_2i4sv_R tumors, but not in the wtHEK293 tumors, which were devoid of CCK_2_R-expression, implying a receptor-mediated process. Uptake in the CCK_2_R-rich stomach was found to be very low and to rapidly decline for all compounds [4]. On the other hand, the background clearance and excretion route differed amongst analogs and radiometals. It is evident that the pendant (radio)metal-chelate has a strong impact not only on receptor affinities and cell uptake but, most importantly, on the tumor-targeting capabilities and overall pharmacokinetic profile of tested GAS1/2/3-radioligands.

Specifically, in the Ga-67 set of compounds, a rank could be established with regards to uptake in the HEK293-CCK_2i4sv_R tumors at all time intervals. For example, for the 1 h pi interval radioligands could be ranked as follows: [^67^Ga]Ga-GAS3 (21.75 ± 4.90%IA/g) > [^67^Ga]Ga-GAS1 (12.62 ± 1.76%IA/g; *p* < 0.0001) > [^67^Ga]Ga-GAS2 (4.06 ± 0.07%IA/g; *p* < 0.0001). Of particular interest is also the fact that tumor levels for [^67^Ga]Ga-GAS3 remained unchanged between 1 and 4 h pi (21.75 ± 4.90%IA/g at 1 h vs. 20.39 ± 3.96%IA/g at 4 h pi; *p* > 0.05). Furthermore, [^67^Ga]Ga-GAS3, was cleared mainly via the kidneys, as opposed to [^67^Ga]Ga-GAS2, which displayed high hepatobiliary excretion with unfavorably high radioactivity levels in the intestines at 1 and 4 h pi (>25%IA/g).

A similar trend was observed in the In-111 set of compounds, as far as the uptake in the HEK293-CCK_2i4sv_R tumors at 4 h pi is concerned, and specifically: GAS3 (19.83 ± 1.35% IA/g) > [^111^In]In-GAS1 (17.79 ± 1.73% IA/g; *p* < 0.01) >> [^111^In]In-GAS2 (8.91 ± 1.59% IA/g; *p* < 0.0001). Differences across radioligands were found more pronounced at 24 h pi, with [^111^In]In-GAS3 (11.53 ± 1.93% IA/g) > [^111^In]In-GAS1 (5.40 ± 1.13% IA/g; *p* < 0.0001) > [^111^In]In-GAS2 (2.78 ± 0.43% IA/g; *p* < 0.0001). Background clearance followed a similar trend to that of Ga-67 analogs. Thus, [^111^In]In-GAS3 was predominantly cleared via the kidneys while [^111^In]In-GAS2 displayed significant intestinal values at 4 h pi (9.71 ± 0.42% IA/g). All three In-111 radioligands displayed notable renal uptake. In a pilot experiment, the kidney protection agent gelofusine was coinjected together with [^111^In]In-GAS1/2 to detect a potential reduction of renal accumulation at both the 4 and 24 h pi intervals, reported previously for [^99m^Tc]Tc-DGA1, [^99m^Tc]Tc-DG2 and many other CCK_2_R-targeting peptide radiotracers [38,44]. Surprisingly, gelofusine failed to reduce kidney uptake or any other pharmacokinetic parameter (Tables S6 and S7; Supplementary File).

Concordant with these findings, [^177^Lu]Lu-GAS3 showed higher HEK293-CCK_2i4sv_R tumor-targeting capacity compared with [^177^Lu]Lu-GAS1 at all time intervals (Tables S9 and S10, respectively; Supplementary File). For example, [^177^Lu]Lu-GAS3 > [^177^Lu]Lu-GAS1 at 4 h pi (20.88 ± 1.20% IA/g vs. 8.74 ± 1.60% IA/g; *p* < 0.0001) and at 24 h pi (5.38 ± 0.77% IA/g vs. 2.09 ± 0.50% IA/g; *p* < 0.0001). However, tumor values significantly declined with time. On the other hand, they both showed rapid background clearance predominantly via the kidneys. The kidneys represented the organ with the highest activity uptake after the HEK293-CCK_2i4sv_R tumors, but renal levels drastically dropped between 4 and 24 h pi (e.g., [^177^Lu]Lu-GAS3 from 8.86 ± 0.67% IA/g to 1.42 ± 0.29% IA/g, respectively; *p* < 0.0001). It should be noted that for [^177^Lu]Lu-GAS3 tumor to kidney favorably increased with time, e.g., from 2.4 at 4 h pi, to 3.8 at 24 h pi and up to 13.8 at 96 pi.

### 3.6. SPECT/CT with [^111^In]In-GAS1/2/3

**Figure 5:**
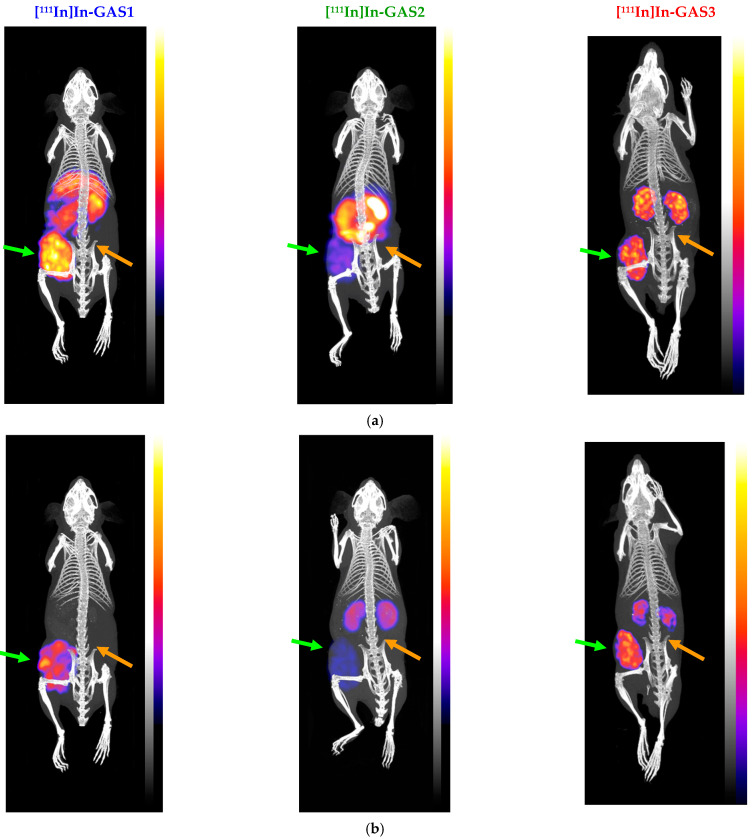
Static whole-body SPECT/CT images of SCID mice bearing twin HEK293-CCK_2i4sv_R and wtHEK293 tumors in their flanks at (**a**) at 4 h pi and (**b**) at 24 h pi of [^111^In]In-GAS1/2/3; green arrows are pointing to HEK293-CCK_2i4sv_R xenografts, and orange arrows are indicating the wtHEK293 tumors. Intense uptake is observed in the CCK_2i4sv_R expressing tumors, but no uptake is evident in the tumors devoid of CCK_2i4sv_R expression. The color bars indicate the difference in accumulated activity (purple being the lowest and white being the highest level of accumulation).

SPECT/CT imaging was performed for [^111^In]In-GAS1/2/3 at 4 and 24 h pi in SCID mice bearing double HEK293-CCK_2i4sv_R and wtKEK293 tumors in their flanks, and the results are summarized in Figure 5.

We observed that radioactivity was selectively taken up only by the HEK293-CCK_2i4sv_R tumors, with the receptor-negative tumors remaining devoid of radioactivity for all compounds. Thus, concordant with biodistribution findings, these results reveal that tumor uptake of [^111^In]In-GAS1/2/3 is a CCK_2i4sv_R-mediated process. Furthermore, and in agreement with biodistribution results, [^111^In]In-GAS3 displayed a superior pharmacokinetic profile.

## 4. Discussion

The development of gastrin-based anti-CCK_2_R radioligands for the theranostic management of human tumors showed significant advances in recent years [8]. Despite this, a few pending issues need to be tackled, for example, the side-effects elicited after CCK_2_R activation by agonists injected to patients [25], or the inadvertent accumulation of radioactivity in tissues with high physiological CCK_2_R-expression (e.g., the gastric mucosa) [11,22,23,42]. Therefore, a shift of paradigm toward radiolabeled CCK_2_R-antagonists represents the next rational step to take in this venture, mimicking recent developments in the fields of somatostatin and bombesin [45,46]. Adopting this concept, we recently introduced [^99m^Tc]Tc-DGA1, a radiotracer based on the nonpeptidic antagonist Z360 and suitable for visualizing CCK_2_R-positive lesions with SPECT/CT [38]. Aiming toward Z360-radioligands with a broader theranostic profile, we now present GAS1/2/3. These Z360-conjugates were modified by three different macrocyclic chelators (DOTA, NODAGA, or DOTAGA) suitable for labeling with trivalent radiometals of clinical interest with each chelator tethered to the free carboxylate group of Z360 via the common linker H-DGlu-HN(PEG3)NH_2_ (Figure 1). Hence, unlike DGA1, GAS1/2/3 lack the (DGlu)_4_-chain implicated in the observed high renal uptake of [^99m^Tc]Tc-DGA1 [38].

The CCK_2_R-affinity of the three bioconjugates was found to be slightly inferior to the unmodified Z360. After the incorporation of gallium, indium or lutetium, more pronounced effects on CCK_2_R-affinity could be observed across analogs, with the poorest affinity displayed by [^nat^In]In-GAS2 (Table 1). Differences in the metal-chelate charge, polarity and overall configuration residing on distinct coordination chemistries of the aforementioned trivalent metals with the three chelators [47] seem to play a role in determining the CCK_2_R-affinity of the resulting metal-tagged compounds. It should be noted that the GAS3 series displayed superior CCK_2_R-affinities compared with the respective GAS1 and GAS2 counterparts.

Next, the GAS1/2/3 were studied for agonism–antagonism at the CCK_2_R applying Ca^2+^ mobilization assays in HEK293-hCCK2R-YC3.60 cells. As expected, the two positive controls [Leu^15^]gastrin-17 and DG2, behaved as typical agonists, triggering Ca^2+^ mobilization at concentrations as low as 100 pM and 10 pM, respectively (Figure S1A,B; Supplementary File). At this concentration level, none of the GAS1/2/3 or the Z360 reference were able to induce a visible effect. Unexpectedly though, these analogs showed agonistic properties at concentrations >100 nM (Figures S1C and S2A–C; Supplementary File). Furthermore, during additional pilot experiments, they failed to inhibit Ca^2+^ mobilization induced by either [Leu^15^]gastrin-17 or DG2 agonists (results not shown), in contrast to DGA1 previously showing a pure antagonist profile at the CCK_2_R [38]. The intriguing behavior of GAS1/2/3 and Z360 at the CCK_2_R seems to be in line with an allosteric modulator profile, as reported for other benzodiazepine analogs [48,49], and requires dedicated studies to be fully explored. For nuclear medicine applications, the absence of CCK_2_R-activation by GAS1/2/3 at concentrations <100 nM is significant and associated with sufficient safety for injection in humans. Notably, hitherto clinical studies, including those using therapeutic doses, involve the administration of CCK_2_R-agonists well below 30 nmol/patient [22,23]. This would translate to a concentration of a maximum of 6 nM in a 75 kg patient (without taking into account the rapid blood clearance or the much higher distribution volume in the patient than the 5 Lt blood volume). This value is well below the threshold of CCK_2_R-activation by any of GAS1/2/3.

Radiolabeled GAS1/2/3 displayed different receptor-specific uptake during incubation in HEK293-CCK_2i4sv_R cells, but in general, cell uptake followed binding affinity patterns. For example, the most affine [^111^In]In-GAS3 displayed the highest cell-uptake values at 1 h and [^111^In]In-GAS2 the poorest (Figure 3 and Table 1). Another interesting feature in the cell uptake of these radioligands is the varying distribution of activity between the internalized and membrane-bound fragments across analogs. Thus, the internalized to membrane-bound ratio was as high as 4.1 for [^111^In]In-GAS3 and 2.8 for [^177^Lu]Lu-GAS3, but dropped to considerably lower values for [^67^Ga]Ga-GAS1 (0.7) and [^67^Ga]Ga-GAS2 (0.9). The observed cell-uptake and internalization pattern is not consistent with a typical receptor antagonist and evokes the unlikely behavior of GAS1/2/3 during the Ca^2+^-mobilization assays. Previous reports showed benzodiazepine analogs behaving as antagonists during Ca^2+^ mobilization functional assays while at the same time inducing partial or full CCK_2_R-internalization in different assays [50,51].

Following injection in healthy mice, the radioligands displayed high metabolic stability in peripheral mice blood at 5 min pi and, with the exception of [^111^In]In-GAS2, were found excreted without further degradation in the urine at 30 min pi (Figure 3 and Table 2). This finding represents an advantageous trait of the new nonpeptidic Z360-radioligands vs. their peptidic counterparts based on gastrin. The latter, in particular the *des*-(Glu)_5_ analogs, were shown to undergo fast enzymatic degradation after entering the bloodstream by NEP and possibly also ACE [11,12,13,14,15,43,52,53]. It should be noted that radiopeptide metabolic stability improvements could be achieved either via in situ NEP and ACE inhibition strategies or after extensive structural interventions of peptide motifs [8].

The tumor-targeting capabilities and overall biodistribution patterns of GAS1/2/3-radioligands were studied in immunosuppressed mice bearing a twin xenograft model, namely subcutaneous tumors from either HEK293-CCK_2i4sv_R or wtHEK293 cells (Figure 4, Tables S3–S9 in Supplementary File). We observed that biodistribution profiles depend on radiometal-chelate, with the targeting of HEK293-CCK_2i4sv_R tumors being superior in the case of Ga-67 and In-111 labeled GAS1 and GAS3, compared with GAS2. Furthermore, [^67^Ga]Ga-GAS2 displayed significantly higher intestinal accumulation vs. both [^67^Ga]Ga-GAS1/3, while [^111^In]In-GAS2 showed unfavorably higher uptake in both the kidneys and intestines vs. the [^111^In]In-GAS1/3 counterparts. Overall, the NODAGA-Z360-based [^67^Ga]Ga/[^111^In]In-radioligands showed the least favorable tumor-to-background profile vs. the DOTA/DOTAGA-modified radioligands. On the contrary, GAS1 and GAS3 displayed high tumor targeting and lower background levels after labeling with either Ga-67, In-111, or Lu-177. Amongst the two, [^67^Ga]Ga/[^111^In]In/[^177^Lu]Lu-GAS3 displayed a superior profile in mice than the corresponding [^67^Ga]Ga/[^111^In]In/[^177^Lu]Lu-GAS1. In the case of the [^111^In]In-GAS1/2/3 radioligands, these results were nicely illustrated by SPECT/CT (Figure 5).

The present study revealed a few promising features with regards to the application of [^177^Lu]Lu-GAS1/3 as anti-CCK_2_R tumor therapeutics. Clearly, washout from the experimental tumors from 4 to 24 h pi is concerning; this uptake gradually declined further at the later time points. However, background activity declined notably faster, resulting in positive tumor-to-background ratios over time, a finding in support of future therapy prospects. Such prospects should be further validated by futuresystematic therapy studies in mice, aiming to address a number of questions still open at this preliminary stage. On the one hand, Z360- conjugate/radionuclide doses and administration schemes need to be explored and optimized to maximize therapeutic efficacy. Then, potential damage to healthy tissues needs to be carefully investigated by dedicated immune-histological assays and reliable dosimetry calculations to be performed before setting toxicity limits. In this respect, the lack of CCK_2_R-activation at concentrations <100 nM, along with the low radioactivity levels of the new analogs in the stomach, represent clear advantages. Carefully conducted therapy studies at the preclinical level will provide crucial information on the therapeutic value of either [^177^Lu]Lu-GAS1/3 per se, but may also hint at the need for further modifications on the radioligand structure (with focus on the linker) or the application of alternative to Lu-177 particle emitters with better suited nuclear properties [47].

Compared with the previously reported biodistribution data of [^99m^Tc]Tc-DGA1 in the same animal model, we observed a much higher tumor to kidney ratio for all [^67^Ga]Ga/[^111^In]In/[^177^Lu]Lu-GAS1/3 radioligands and an overall improvement of the pharmacokinetic profiles [38]. Of particular interest is the fact that mice treatment with gelofusine, previously shown to significantly reduce the kidney accumulation of [^99m^Tc]Tc-DGA1 (from 96.09 ± 12.01% IA/g to 33.61 ± 2.67% IA/g at 4 h pi; *p* < 0.0001), had no effect on the renal uptake of [^111^In]In-GAS1 (6.06 ± 1.24% IA/g vs. gelofusine 5.93 ± 0.56% IA/g at 4 h pi; *p* > 0.05) or [^111^In]In-GAS2 (29.55 ± 3.64% IA/g vs. gelofusine 21.90 ± 3.97% IA/g at 4 h pi; *p* > 0.05), correlating the effect of the kidney protection agent with the (DGlu)_4_-chain in [^99m^Tc]Tc-DGA1.

Another quite attractive feature of radiolabeled GAS1/2/3, in line with a radioantagonist profile, is the low uptake in the CCK_2_R-rich stomach. As mentioned above, this feature becomes particularly important for therapeutic purposes due to dosimetric restrictions [8]. Interestingly, the injection of higher peptide amounts was shown to reduce stomach uptake in preclinical models, but it is associated with more biosafety risks in the case of CCK_2_R-agonists [54].

Although a hitherto restricted number of other Z360 analogs carrying the DOTA-chelator through different linkers was developed [55], animal data was only reported for [^111^In]In-IP-001, but in a mice model different to ours [56]. Thus, an A549 non-small cell lung cancer xenograft was implanted in the shoulder of homozygous female BALB/c nude mice. Specific but low uptake could be established in the tumor on SPECT/CT 4 and 24 h after injection of the radioligand combined with an unfavorably high radioactivity background compared with the scans of Figure 5 and the biodistribution results of this study, especially for the GAS3 (and to a lesser extent the GAS1) radioligand series.

## 5. Conclusions

In the present work, three new bioconjugates of the benzodiazepine-like CCK_2_R-antagonist Z360 were introduced, each coupled with one of the DOTA, NODAGA and DOTAGA chelators via a common linker, and amenable for stable labeling with clinically interesting trivalent radiometals, such as Ga-67 (surrogate for the PET radiometal Ga-68), In-111 and Lu-177 included in our study, and combining several attractive features. Firstly, they displayed receptor affinities dependent upon the metal-chelate, with a few members showing single-digit nM receptor affinities. During functional assays at the cellular level, none of the bioconjugates activated the CCK_2_R at clinically relevant concentrations, thereby providing a dose-safety window for future human applications. The nonpeptidic Z360-radioconjugates tested herein displayed excellent in vivo stability in mice, as opposed to their gastrin-based peptidic counterparts undergoing fast in vivo degradation by NEP and sometimes also ACE. As a result, the new Z360 radioligands were able to specifically target CCK_2_R-positive xenografts in mice but not CCK_2_R-negative control tumors and displayed a low uptake in the CCK_2_R-rich stomach, as consistent with receptor radioantagonists. The background clearance was fast, especially for the GAS3 (DOTAGA-carrying member) and to a lesser extent for the GAS1 (DOTA-carrying member) radioligand series. These excellent qualities confirm previous experience with somatostatin and bombesin antagonist radioligands, paving the way for the first therapy and clinical translation studies of CCK_2_R-antagonists in MTC and other tumor patients.

## Figures and Tables

**Table 1:** Receptor affinities Z360 and its analogs as determined during competition assays against [^125^I][I-Tyr^12^,Leu^15^]gastrin-17 in HEK293-CCK_2i4sv_R cell membranes.

Compound	Z360	GAS1	GAS2	GAS3
metal-free	1.2 ± 0.5 (5)	5.9 ± 1.8 (3)	4.1 ± 1.1 (3)	3.1 ± 1.5 (3)
^nat^Ga	na ^1^	13.8 ± 2.7 (3)	8.8 ± 1.6 (3)	4.8 ± 0.6 (3)
^nat^In	na ^1^	5.0 ± 1.1 (3)	53.2 ± 2.7 (3)	1.9 ± 0.2 (3)
^nat^Lu	na ^1^	12.4 ± 0.2 (3)	na ^1^	4.3 ± 0.9 (3)

^1^ non-applicable; results are expressed in nM and represent mean IC_50_ values ± SD with the number of experiments shown in parentheses.

**Table 2:** In vivo degradation of radioligands in mice blood and urine.

Radiometal Chelate/Analog		GAS1	GAS2	GAS2
[^67^Ga]Ga	Blood ^1^	93.9 ± 0.6 (3)	96.3 ± 3.7 (3)	92.7 ± 0.7 (3)
	Urine ^2^	80.2 ± 1.0 (3)	81.6 ± 1.5 (3)	78.3 ± 1.5 (3)
[^111^In]In	Blood	84.6 ± 2.4 (3)	73.1 ± 1.8 (3)	98.3 ± 0.3 (3)
	Urine	85.2 ± 5.2 (3)	44.1 ± 2.6 (3)	94.3 ± 1.3 (3)
[^177^Lu]Lu	Blood	81.1 ± 8.2 (3)	-	95.8 ± 2.0 (3)
	Urine	79.2 ± 10.3 (3)	-	88.1 ± 0.4 (3)

^1^ Blood was collected 5 min pi; ^2^ Urine was collected 30 min pi; results were expressed as a percentage of intact radioligand and represent mean values ± SD, with the number of independent experiments shown in parentheses.

## Data Availability

The data presented in this study is available in this article (and Supplementary Material).

## Supplementary Materials

The following supporting information can be downloaded at: https://www.mdpi.com/article/10.3390/pharmaceutics14030666/s1, Analytical data for GAS1/2/3, Table S1: Analytical data for GAS1/2/3; Labeling of GAS-compounds with Ga-67, In-111 and Lu-177; Preparation of [^nat^Ga]Ga-GAS1/2/3, [^nat^In]In-GAS1/2/3 and [^nat^Lu]Lu-GAS1/3; Table S2. Analytical HPLC data for (radio)metal-tagged GAS1/2/3; Ca^2+^ mobilization assays; Figure S1: Ca^2^+ mobilization assays for [Leu15]gastrin-17, DG2, and Z360 references in HEK293 cells contingent on the expression of CCK_2_R; Figure S2: Ca^2+^ mobilization assays for GAS1/2/3 in HEK293 cells contingent on the expression of CCK_2_R; Tables S3–S10: Biodistribution results for GAS1/2/3 labeled with Ga-67, In-111 and Lu-177 in SCID mice bearing double HEK293-CCK_2i4sv_R/wtHEK293 tumors. References [57,58] are cited in the supplementary materials.

## References

[1] Reubi J.C., Schaer J.C., Waser B. (1997). Cholecystokinin(CCK)-A and CCK-B/gastrin receptors in human tumors. Cancer Res..

[2] Reubi J.C., Waser B. (1996). Unexpected high incidence of cholecystokinin-B/gastrin receptors in human medullary thyroid carcinomas. Int. J. Cancer.

[3] Ferrand A., Wang T.C. (2006). Gastrin and cancer: A review. Cancer Lett..

[4] Dufresne M., Seva C., Fourmy D. (2006). Cholecystokinin and gastrin receptors. Physiol. Rev..

[5] Reubi J.C. (2001). CCK receptors in human neuroendocrine tumors: Clinical implications. Scand. J. Clin. Lab. Investig..

[6] Gotthardt M., Béhé M.P., Grass J., Bauhofer A., Rinke A., Schipper M.L., Kalinowski M., Arnold R., Oyen W.J.G., Behr T.M. (2006). Added value of gastrin receptor scintigraphy in comparison to somatostatin receptor scintigraphy in patients with carcinoids and other neuroendocrine tumours. Endocr. Relat. Cancer.

[7] Béhé M., Behr T.M. (2002). Cholecystokinin-B (CCK-B)/gastrin receptor targeting peptides for staging and therapy of medullary thyroid cancer and other CCK-B receptor expressing malignancies. Biopolymers.

[8] von Guggenberg E., Kolenc P., Rottenburger C., Mikolajczak R., Hubalewska-Dydejczyk A. (2021). Update on preclinical development and clinical translation of cholecystokinin-2 receptor targeting radiopharmaceuticals. Cancers.

[9] Nock B.A., Maina T., Krenning E.P., de Jong M. (2014). “To Serve and Protect”: Enzyme Inhibitors as Radiopeptide Escorts Promote Tumor Targeting. J. Nucl. Med..

[10] Laverman P., Joosten L., Eek A., Roosenburg S., Peitl P.K., Maina T., Mäcke H., Aloj L., Von Guggenberg E., Sosabowski J.K. (2011). Comparative biodistribution of 12 111In-labelled gastrin/CCK2 receptor-targeting peptides. Eur. J. Pediatr..

[11] Fröberg A.C., De Jong M., Nock B.A., Breeman W.A.P., Erion J.L., Maina T., Verdijsseldonck M., De Herder W.W., Van Der Lugt A., Kooij P.P.M. (2009). Comparison of three radiolabelled peptide analogues for CCK-2 receptor scintigraphy in medullary thyroid carcinoma. Eur. J. Pediatr..

[12] Kaloudi A., Nock B.A., Lymperis E., Krenning E.P., De Jong M., Maina T. (2016). Improving the In Vivo Profile of Minigastrin Radiotracers: A Comparative Study Involving the Neutral Endopeptidase Inhibitor Phosphoramidon. Cancer Biother. Radiopharm..

[13] Kaloudi A., Nock B.A., Lymperis E., Krenning E.P., de Jong M., Maina T. (2016). ^99m^Tc-Labeled gastrins of varying peptide chain length: Distinct impact of NEP/ACE-inhibition on stability and tumor uptake in mice. Nucl. Med. Biol..

[14] Kaloudi A., Nock B.A., Lymperis E., Sallegger W., Krenning E.P., de Jong M., Maina T. (2015). In vivo inhibition of neutral endo-peptidase enhances the diagnostic potential of truncated gastrin ^111^In-radioligands. Nucl. Med. Biol..

[15] Kaloudi A., Nock B.A., Lymperis E., Valkema R., Krenning E.P., De Jong M., Maina T. (2016). Impact of clinically tested NEP/ACE inhibitors on tumor uptake of [^111^In-DOTA]MG11—First estimates for clinical translation. EJNMMI Res..

[16] Valkema R., Fröberg A., Maina T., Nock B.A., de Blois E., Melis M., Konijnenberg M.W., Koolen S.L.W., Peeters R.P., de Herder W.W. (2019). Clinical translation of the PepProtect concept: Improved detection of cancer and metastases, applied in medullary thyroid cancer patients with [^111^In]In-MG11 scanning during neprilysin inhibition. Eur. J. Nucl. Med. Mol. Imaging.

[17] Maina T., Konijnenberg M.W., Kolenc-Peitl P., Garnuszek P., Nock B.A., Kaloudi A., Kroselj M., Zaletel K., Maecke H., Mansi R. (2016). Preclinical pharmacokinetics, biodistribution, radiation dosimetry and toxicity studies required for regulatory approval of a phase I clinical trial with ^111^In-CP04 in medullary thyroid carcinoma patients. Eur. J. Pharm. Sci..

[18] Kolenc-Peitl P., Mansi R., Tamma M., Gmeiner-Stopar T., Sollner-Dolenc M., Waser B., Baum R.P., Reubi J.C., Maecke H.R. (2011). Highly Improved Metabolic Stability and Pharmacokinetics of Indium-111-DOTA-Gastrin Conjugates for Targeting of the Gastrin Receptor. J. Med. Chem..

[19] Corlett A., Sani M.-A., Van Zuylekom J., Ang C.-S., von Guggenberg E., Cullinane C., Blyth B., Hicks R.J., Roselt P.D., E Thompson P. (2021). A New Turn in Peptide-Based Imaging Agents: Foldamers Afford Improved Theranostics Targeting Cholecystokinin-2 Receptor-Positive Cancer. J. Med. Chem..

[20] Uprimny C., Bayerschmidt S., di Santo G., Klingler M., Hormann A., Warwitz B., Rangger C., von Guggenberg E., Virgolini I. (2021). First results of biodistribution and tumour targeting of ^68^Ga-DOTA-MGS5 PET/CT in advanced medullary thyroid cancer patients. Eur. J. Nucl. Med. Mol. Imaging.

[21] Grob N., Häussinger D., Deupi X., Schibli R., Behe M., Mindt T.L. (2020). Triazolo-Peptidomimetics: Novel Radiolabeled Minigastrin Analogs for Improved Tumor Targeting. J. Med. Chem..

[22] Erba P.A., Maecke H., Mikolajczak R., Decristoforo C., Zaletel K., Maina-Nock T., Peitl P.K., Garnuszek P., Fröberg A., Goebel G. (2018). A novel CCK2/gastrin receptor-localizing radiolabeled peptide probe for personalized diagnosis and therapy of patients with progressive or metastatic medullary thyroid carcinoma: A multicenter phase I GRAN-T-MTC study. Pol. Arch. Intern. Med..

[23] Rottenburger C., Nicolas G.P., McDougall L., Kaul F., Cachovan M., Vija A.H., Schibli R., Geistlich S., Schumann A., Rau T. (2020). Cholecystokinin 2 Receptor Agonist ^177^Lu-PP-F11N for Radionuclide Therapy of Medullary Thyroid Carcinoma: Results of the Lumed Phase 0a Study. J. Nucl. Med..

[24] Uprimny C., von Guggenberg E., Svirydenka A., Mikolajczak R., Hubalewska-Dydejczyk A., Virgolini I.J. (2021). Comparison of PET/CT imaging with [^18^F]FDOPA and cholecystokinin-2 receptor targeting [^68^Ga]Ga-DOTA-MGS5 in a patient with advanced medullary thyroid carcinoma. Eur. J. Nucl. Med. Mol. Imaging.

[25] Ubl P., Gincu T., Keilani M., Ponhold L., Crevenna R., Niederle B., Hacker M., Li S. (2014). Comparison of side effects of pentagastrin test and calcium stimulation test in patients with increased basal calcitonin concentration: The gender-specific differences. Endocrine.

[26] Grzmil M., Imobersteg S., Blanc A., Frank S., Schibli R., Béhé M.P. (2021). Therapeutic Response of CCKBR-Positive Tumors to Combinatory Treatment with Everolimus and the Radiolabeled Minigastrin Analogue [^177^Lu]Lu-PP-F11N. Pharmaceutics.

[27] Novak D., Anderluh M., Peitl P.K. (2020). CCK_2_R antagonists: From SAR to clinical trials. Drug Discov. Today.

[28] Morita H., Miura N., Hori Y., Matsunaga Y., Ukawa H., Suda H., Yoneta T., Kurimoto T., Itoh Z. (2001). Effects of Z-360, a novel CCKB/gastrin (CCK2) receptor antagonist, on meal-induced acid secretion and experimental ulcer models in dogs and rats. Gastroenterology.

[29] Miura N., Yoneta T., Ukawa H., Fukuda Y., Eta R., Mera Y., Omata T., Kinomoto T., Kurimoto T., Itoh Z. (2001). Pharmacological profiles of Z-360, a novel CCKB/gastrin (CCK2) receptor antagonist with excellent oral potency. Gastroenterology.

[30] Ukawa H., Miura N., Morita H., Hori Y., Ueki S., Yoneta T., Kurimoto T., Itoh Z. (2002). Effect of Z-360, a selective CCKB/gastrin receptor antagonist, on chronic acid reflux esophagitis in rats. Gastroenterology.

[31] Grabowska A., Morris T., McKenzie A., Kumari R., Hamano H., Emori Y., Yoshinaga K., Watson S. (2008). Pre-clinical evaluation of a new orally-active CCK-2R antagonist, Z-360, in gastrointestinal cancer models. Regul. Pept..

[32] Kawasaki D., Emori Y., Eta R., Iino Y., Hamano H., Yoshinaga K., Tanaka T., Takei M., Watson S.A. (2008). Effect of Z-360, a novel orally active CCK-2/gastrin receptor antagonist on tumor growth in human pancreatic adenocarcinoma cell lines in vivo and mode of action determinations in vitro. Cancer Chemother. Pharm..

[33] Meyer T., Caplin M., Palmer D., Valle J., Larvin M., Waters J., Coxon F., Borbath I., Peeters M., Nagano E. (2010). A phase Ib/IIa trial to evaluate the CCK2 receptor antagonist Z-360 in combination with gemcitabine in patients with advanced pancreatic cancer. Eur. J. Cancer.

[34] Ueno M., Li C.P., Ikeda M., Ishii H., Mizuno N., Yamaguchi T., Ioka T., Oh D.Y., Ichikawa W., Okusaka T. (2017). A randomized phase II study of gemcitabine plus Z-360, a CCK2 receptor-selective antagonist, in patients with metastatic pancreatic cancer as compared with gemcitabine plus placebo. Cancer Chemother. Pharmacol..

[35] Wayua C., Roy J., Putt K.S., Low P.S. (2015). Selective Tumor Targeting of Desacetyl Vinblastine Hydrazide and Tubulysin B via Conjugation to a Cholecystokinin 2 Receptor (CCK_2_R) Ligand. Mol. Pharm..

[36] Wayua C., Low P.S. (2013). Evaluation of a Cholecystokinin 2 Receptor-Targeted Near-Infrared Dye for Fluorescence-Guided Surgery of Cancer. Mol. Pharm..

[37] Wayua C., Low P.S. (2015). Evaluation of a Nonpeptidic Ligand for Imaging of Cholecystokinin 2 Receptor–Expressing Cancers. J. Nucl. Med..

[38] Kaloudi A., Kanellopoulos P., Radolf T., Chepurny O.G., Rouchota M., Loudos G., Andreae F., Holz G.G., Nock B.A., Maina T. (2020). [^99m^Tc]Tc-DGA1, a Promising CCK_2_R-Antagonist-Based Tracer for Tumor Diagnosis with Single-Photon Emission Computed Tomography. Mol. Pharm..

[39] Körner M., Waser B., Reubi J.-C., Miller L.J. (2010). CCK2 receptor splice variant with intron 4 retention in human gastrointestinal and lung tumours. J. Cell Mol. Med..

[40] Chao C., Han X., Ives K., Park J., Kolokoltsov A.A., Davey R., Moyer M.P., Hellmich M.R. (2009). CCK2 receptor expression transforms non-tumorigenic human NCM356 colonic epithelial cells into tumor forming cells. Int. J. Cancer.

[41] Laverman P., Roosenburg S., Gotthardt M., Park J., Oyen W.J.G., De Jong M., Hellmich M.R., Rutjes F.P.J.T., Van Delft F.L., Boerman O.C. (2007). Targeting of a CCK2 receptor splice variant with ^111^In-labelled cholecystokinin-8 (CCK8) and ^111^In-labelled minigastrin. Eur. J. Pediatr..

[42] Nock B.A., Maina T., Béhé M., Nikolopoulou A., Gotthardt M., Schmitt J.S., Behr T.M., Mäcke H.R. (2005). CCK-2/gastrin receptor-targeted tumor imaging with ^99m^Tc-labeled minigastrin analogs. J. Nucl. Med..

[43] Kanellopoulos P., Kaloudi A., Rouchota M., Loudos G., de Jong M., Krenning E.P., Nock B.A., Maina T. (2020). One step closer to clinical translation: Enhanced tumor targeting of [^99m^Tc]Tc-DB4 and [^111^In]In-SG4 in mice treated with Entresto. Pharmaceutics.

[44] Gotthardt M., van Eerd-Vismale J., Oyen W.J., de Jong M., Zhang H., Rolleman E., Maecke H.R., Béhé M., Boerman O. (2007). Indication for different mechanisms of kidney uptake of radiolabeled peptides. J. Nucl. Med..

[45] Mansi R., Fani M. (2019). Design and development of the theranostic pair ^177^Lu-OPS201/^68^Ga-OPS202 for targeting somatostatin receptor expressing tumors. J. Labelled Comp. Radiopharm..

[46] Maina T., Nock B.A., Kulkarni H., Singh A., Baum R.P. (2017). Theranostic prospects of gastrin-releasing peptide receptor-radioantagonists in oncology. PET Clin..

[47] Kostelnik T.I., Orvig C. (2019). Radioactive Main Group and Rare Earth Metals for Imaging and Therapy. Chem. Rev..

[48] Schwartz T.W., Holst B. (2007). Allosteric enhancers, allosteric agonists and ago-allosteric modulators: Where do they bind and how do they act?. Trends Pharmacol. Sci..

[49] Desai A.J., Henke B.R., Miller L.J. (2015). Elimination of a cholecystokinin receptor agonist ‘trigger’ in an effort to develop positive allosteric modulators without intrinsic agonist activity. Bioorganic Med. Chem. Lett..

[50] Roettger B.F., Ghanekar D., Rao R., Toledo C., Yingling J., Pinon D., Miller L.J. (1997). Antagonist-stimulated internalization of the G protein-coupled cholecystokinin receptor. Mol. Pharmacol..

[51] Akgün E., Körner M., Gao F., Harikumar K.G., Waser B., Reubi J.C., Portoghese P.S., Miller L.J. (2009). Synthesis and in Vitro Characterization of Radioiodinatable Benzodiazepines Selective for Type 1 and Type 2 Cholecystokinin Receptors. J. Med. Chem..

[52] Deschodt-Lanckman M., Pauwels S., Najdovski T., Dimaline R., Dockray G. (1988). In vitro and in vivo degradation of human gastrin by endopeptidase 24.11. Gastroenterology.

[53] Dubreuil P., Fulcrand P., Rodriguez M., Fulcrand H., Laur J., Martinez J. (1989). Novel activity of angiotensin-converting enzyme. Hydrolysis of cholecystokinin and gastrin analogues with release of the amidated C-terminal dipeptide. Biochem. J..

[54] Klingler M., Hörmann A.A., Rangger C., Desrues L., Castel H., Gandolfo P., Von Guggenberg E. (2020). Stabilization Strategies for Linear Minigastrin Analogues: Further Improvements via the Inclusion of Proline into the Peptide Sequence. J. Med. Chem..

[55] Novak D., Tomašič T., Krošelj M., Javornik U., Plavec J., Anderluh M., Peitl P.K. (2021). Radiolabelled CCK_2_R Antagonists Containing PEG Linkers: Design, Synthesis and Evaluation. ChemMedChem.

[56] Verona M., Rubagotti S., Croci S., Sarpaki S., Borgna F., Tosato M., Vettorato E., Marzaro G., Mastrotto F., Asti M. (2021). Preliminary Study of a 1,5-Benzodiazepine-Derivative Labelled with Indium-111 for CCK-2 Receptor Targeting. Molecules.

[57] Milliken B.T., Doyle R.P., Holz G.G., Chepurny O.G. (2020). FRET reporter assays for cAMP and calcium in a 96-well format using genetically encoded biosensors expressed in living cells. Bio-Protocol.

[58] Chepurny O.G., Matsoukas M.T., Liapakis G., Leech C.A., Milliken B.T., Doyle R.P., Holz G.G. (2019). Nonconventional glucagon and GLP-1 receptor agonist and antagonist interplay at the GLP-1 receptor revealed in high-throughput FRET assays for cAMP. J. Biol. Chem..

