# Supplementary material for: Nonpeptidic Z360-Analogs Tagged with Trivalent Radiometals as Anti-CCK2R Cancer Theranostic Agents: A Preclinical Study
Source: Pharmaceutics. 2022 Mar 18;14(3):666. doi: 10.3390/pharmaceutics14030666 (PMC8954547; doi:10.3390/pharmaceutics14030666)
Supplement: Supplementary file 1 [file pharmaceutics-14-00666-s001.zip › pharmaceutics-1642341-SM.pdf]

pharmaceutics

MDPI

Supplementary Materials: Nonpeptidic Z360-Analogs Tagged with Trivalent Radiometals as Anti-CCK2R Cancer Theranostic Agents: A Preclinical Study

Berthold A. Nock, Panagiotis Kanellopoulos, Oleg G. Chepurny, Maritina Rouchota, George Loudos, George G. Holz, Eric P. Krenning and Theodosia Maina

Analytical data for GAS1/2/3

Analytical data from the synthesis of GAS1/2/3, comprising results from MALDI-TOF mass spectroscopy and from HPLC analysis in two distinct HPLC systems, were compiled in Table S1.

Table S1. Analytical Data for GAS1/2/3.

compound	structure	% purity[a]	MW[b] calcd MW[b] found, m/z	HPLC t_R (min) UV trace	
				system 1[c]	system 2[d]
**GAS1**	**DOTA-DGlu-HN(PEG)3NH-Z360**	**≥ 95**	1210.4/1210.9, [M + H]$^+$	27.0	17.0
**GAS2**	NODAGA-DGlu-HN(PEG)3NH-Z360	≥ 95	1181.3/1181.9, [M + H]$^+$	36.1	17.6
**GAS3**	DOTAGA-DGlu-HN(PEG)3NH-Z360	≥ 95	1282.4//1283.0, [M + H]$^+$	29.0	24.5[e]

[a]Purity was determined by HPLC systems 1 and 2. [b]Average mass. [c]system 1: RP-HPLC with UV detection at 220 nm; a Symmetry Shield RP18 column (5 μm, 3.9 mm × 150 mm) was eluted at a flow rate of 1 mL/min with linear gradient: 100%A/0% B to 70%A/30% B in 5 min and then 70%A/30% B to 55%A/45% B in 60 min, with A = 0.1% TFA in H_2O (v/v) and B = MeCN. [d]system 2: A Nucleosil-100 C18 column (5 μm, 150 mm × 4 mm) was eluted at a flow rate of 1 mL/min with a linear gradient: 0.1 % TFA in MeCN (10% to 90% in 15 min) and 0.1 % aqueous TFA as complementary phase; runs were monitored by UV detection at 215 nm; [e]system 2 modified as: 10% to 90% in 50 min.

Labeling of GAS-compounds with Ga-67, In-111 and Lu-177

Labeling with Ga-67. To an Eppendorf Protein LoBind® centrifuge tube (capacity: 1.5 mL) the following items were successively added: EtOH (40 μL), [^{67}Ga]$GaCl_3$ (27 μL; 4 – 5.5 GBq/mL in dilute HCl), a freshly prepared solution of sodium para-aminobenzoic acid (Na-PABA, 10 μL, 0.5 M) in bi-distilled water, the corresponding GAS1/2/3 precursor stock solution (10 μL, 20 μg, ≈10 nmol) and sodium acetate solution (10 μL, pH 8, 1 M). The mixture was incubated for 30 min at 80 °C (for GAS1 and GAS3) or 40 °C (for GAS2). For quality control a 2 μL aliquot of the labeling solution was withdrawn and quenched with 28 μL of an acetate buffered solution of disodium ethylenediaminetetraacetic acid (Na_2-EDTA, 1 mM, pH 4.0). The [^{67}Ga]Ga-GAS1/2/3 radioligands were produced in radiochemical purities generally exceeding 95% at apparent molecular activities of 10 – 15 MBq [^{67}Ga]Ga/nmol GAS conjugate.

Labeling with In-111. Into an Eppendorf Protein LoBind® centrifuge tube (capacity: 1.5 mL) the following items were successively pipetted: EtOH (100 μL), [^{111}In]$InCl_3$ (150 μL, 400 – 600 MBq/mL in 0.05 M HCl) and a freshly prepared Na-PABA solution (25 μL, 0.5 M). After adding the corresponding GAS conjugate stock solution (25 μL, 50 μg, ≈25 nmol) and sodium acetate buffer (15 μL, pH 4.0, 1 M) the labeling reaction mixture was incubated as follows: GAS1 – at 50 °C for 70 min; GAS2 – at 50 °C for 50 min; GAS3 – at 80 °C for 30 min. Quality control was performed after adding octadentate DTPA as a scavenger of unbound In-111. The [^{111}In]In-GAS1/2/3 radioligands were obtained in radiochemical purities exceeding 95 % at apparent molecular activities of 2.4 – 3.6 MBq [^{111}In]In/nmol GAS conjugate.

Labeling with Lu-177. For labeling of GAS1/3 with Lu-177, the following reagent solutions were pipetted into an Eppendorf Protein LoBind® centrifuge tube (capacity: 1.5 mL): EtOH (120 μL), [^{177}Lu]$LuCl_3$ (150 μL, 3.7 GBq/mL in 0.04 M HCl, A_s > 370 GBq/mg Lu), sodium acetate buffer (30 μL pH 4.6, 1.0 M), a freshly prepared Na-PABA solution (30 μL, 0.5 M) and each of the GAS1/3 stock solution (35 μL, 70 μg, ≈35 nmol). The mixture was heated at 80 °C for 20 min. Quality control was performed after adding octadentate DTPA as a scavenger of unbound Lu-177. [^{177}Lu]Lu-GAS1/3 were produced in high radiochemical purities (>95 %) at apparent molar activities of approximately 16 MBq [^{177}Lu]Lu/nmol ligand at the end of synthesis.

Results from radio-HPLC analysis applying system 1 were compiled in Table S2. In view of the high purity formation of radioligands, no further purification was required for subsequent biological testing. For all further *in vitro* and *in vivo* studies the labeling solution was diluted to the desired activity concentration using a 5 mM solution of Na-PABA dissolved in phosphate buffered saline. The integrity of radiotracers was tested before and after the conclusion of all biological experiments.

Table S2. Analytical HPLC Data for (Radio)Metal-Tagged GAS1/2/3 (t_R (min) gamma/UV trace).

compound	-	[^{67}Ga]Ga/[^{nat}Ga]Ga	[^{111}In]In/[^{nat}In]In	[^{177}Lu]Lu/[^{nat}Lu]Lu
**GAS1**	27.0	30.0	31.7	33.1
**GAS2**	36.1	36.3	34.8	-
**GAS3**	29.0	30.3	36.2	39.3

Purity as determined by HPLC exceeded 97% in all cases. system 1 was applied: RP-HPLC with twin UV detection at 220 nm and gamma detection; a Symmetry Shield RP18 column (5 μm, 3.9 mm × 150 mm) was eluted at a flow rate of 1 mL/min with linear gradient: 100%A/0% B to 70%A/30% B in 5 min and then 70%A/30% B to 55%A/45% B in 60 min, with A = 0.1% TFA in H_2O (v/v) and B = MeCN.

Preparation of [^{nat}Ga]Ga-GAS1/2/3, [^{nat}In]In-GAS1/2/3 and [^{nat}Lu]Lu-GAS1/3

To an Eppendorf Protein LoBind® centrifuge tube containing GAS1/2/3 precursor stock solution (60 μL, 120 μg, ≈60 nmol), a stock solution (60 μL, 2 mM, 120 nmol) of the respective nitrate salt dissolved in 1 M sodium acetate buffer of pH 4.0 (for gallium), 4.6 (for indium) and 5.0 (for lutetium) was added. The mixture was heated at 75 ^{0}C for 1 h. Complete metal incorporation by GAS1/2/3 was revealed by RP-HPLC analysis of the reaction mixture using HPLC system 1, allowing for base line separation of metal-tagged from metal-free GAS1/2/3. Retention times (UV trace, t_R in min) of all GAS species were included in Table S2. The metal-tagged GAS solutions were used as such in *in vitro* competition binding assays assuming a 0.5 mM CCK_2R-ligand concentration.

Ca^{2+} Mobilization Assays

Methods for FRET-based detection of Ca^{2+} in HEK293 cells were described previously [1]. The clone #21 HEK293 cell line (HEK293-hCCK2R/YC3.60) stably co-expressing the human CCK_2R and the Ca^{2+} biosensor YC3.60 was generated according to published methods [2]. The clone #5 HEK293 cell line (HEK293-YC3.60) that expressed only YC3.60 served as a negative control and was generated in a similar matter. The live-cell Ca^{2+} assay was performed in a 96-well format using suspensions of HEK293-hCCK_2R/YC3.60 cells to which test compounds were administered by automated injection using a Flexstation 3 microspectrofluorimeter (Molecular Devices Corp.). The human CCK_2R (NM_176875) in pCMV6-Entry vector contained the receptor's complete open reading frame with a Myc-DDK epitope tag (OriGene Technologies; Cat. No. R200676). Test compounds included human [Leu^{15}]-gastrin-17 (BACHEM Cat. No. 4011661), Z360 (MedChem Express Cat. No. HY-17617), Demogastin-2 (DG2) and GAS1/2/3 (PiChem Forschungs- und Entwickungs GmbH, Raaba-Grambach, Austria). ADP (Sigma-Aldrich) served as a positive control test compound for intracellular Ca^{2+} mobilization.

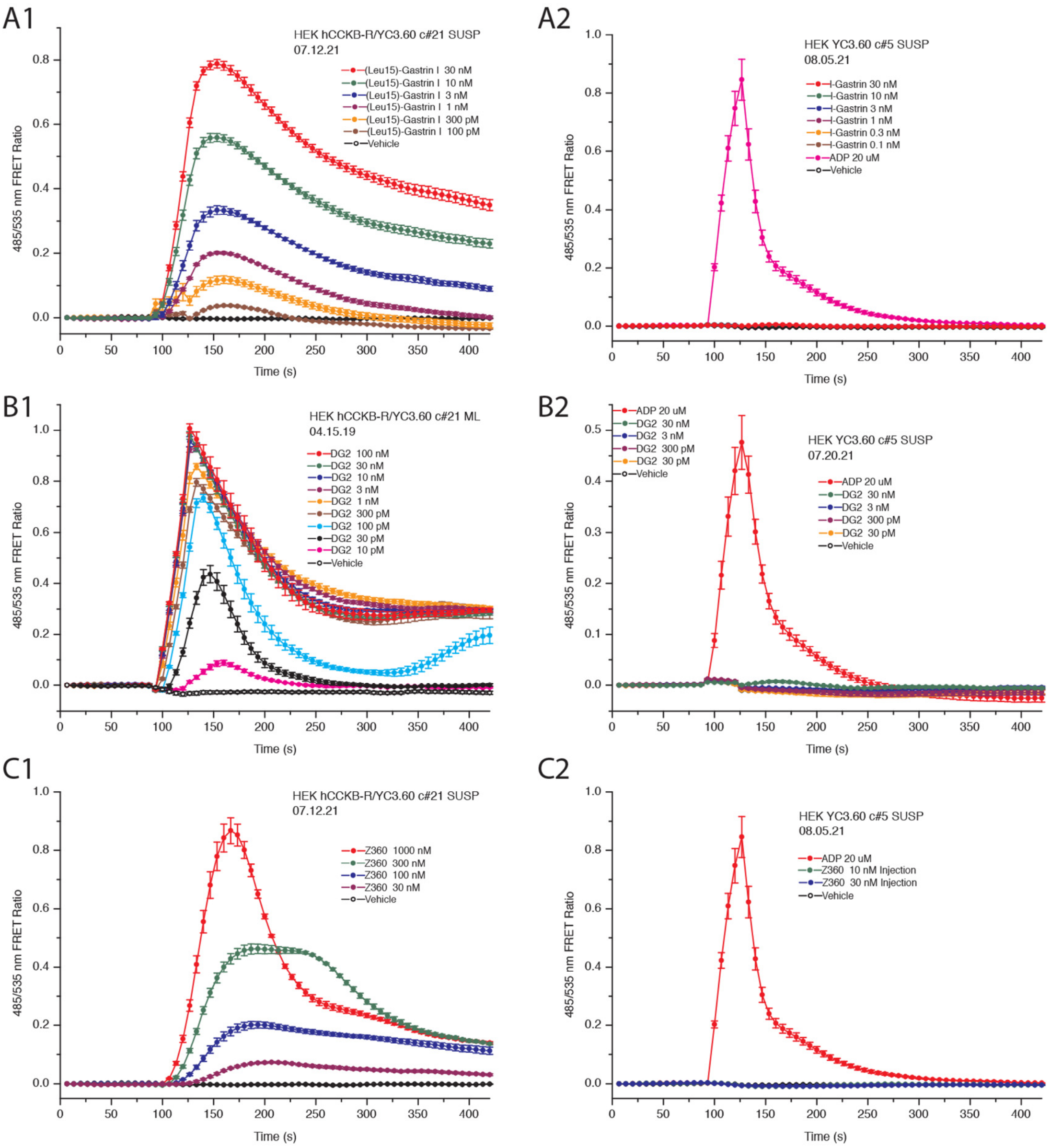

Figure S1. Illustrated are concentration-response relationships for test compounds obtained in YC3.60 FRET-based Ca^{2+} mobilization assays using clone #21 HEK293-hCCK2R-YC3.60 cells (A1, B1, C1) or clone #5 HEK293-YC3.60 cells that do not express CCK_2R (A2, B2, C2). Panels A1 and A2 demonstrate that a Ca^{2+} mobilizing action of $[Leu^{15}]$gastrin-17 was observed using clone #21 but not clone #5 cells. Note that although clone #5 cells failed to respond to $[Leu^{15}]$gastrin-17, they instead responded to ADP that stimulated endogenous purinergic receptors expressed on HEK293 cells. Panels B1 and B2 illustrate findings obtained when testing DG2, whereas panels C1 and C2 illustrate findings obtained when testing Z360. Collectively, these findings demonstrate that the Ca^{2+} mobilizing actions of $[Leu^{15}]$gastrin-17, DG2, and Z360 in HEK293 cells are contingent on expression of CCK_2R.

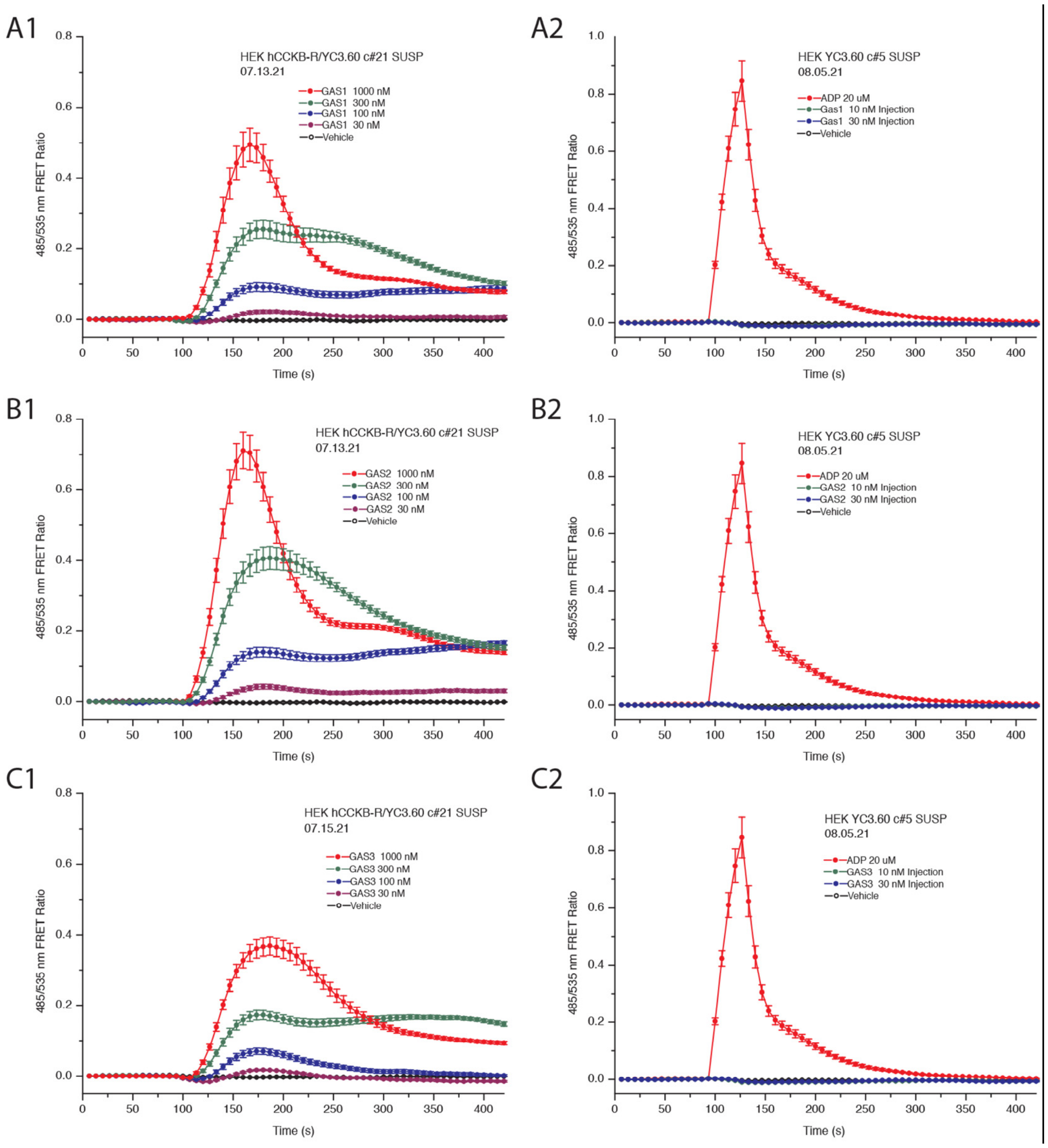

Figure S2. Illustrated are concentration-response relationships for test compounds obtained in assays using clone #21 HEK293-hCCK2R-YC3.60 cells (A1, B1, C1) or clone #5 HEK293-YC3.60 cells (A2, B2, C2). Panels A1 and A2 demonstrate a Ca^{2+} mobilizing action of GAS1 using clone #21 cells but not clone #5 cells. Thus, the agonist action of GAS1 was contingent on expression of CCK_2R. Similar findings were obtained when testing GAS2 (B1, B2) or GAS3 (C1, C2).

Biodistribution in Mice Bearing Twin HEK293-$CCK_{2i4sv}R$ and wtHEK293 Xenografts

Biodistribution results for GAS1/2/3 labeled with Ga-67, In-111 and Lu-177 in SCID mice bearing double HEK293-$CCK_{2i4sv}R$ / wtHEK293 tumors, expressed as %IA/g and representing average values ± SD, n= 4, per animal group, were included in the following Tables S3-S10.

Table S3. Biodistribution of [^{67}Ga]Ga-GAS1 in male SCID mice bearing twin HEK293-CCK$_{2i4sv}$R and wtHEK293 tumors at 1, 4 and 24 h pi, expressed as %IA/g and representing mean values ± SD, n=4.

**Organs**	**%IA/g ± SD, n=4**		
	**1 h**	**4 h**	**24 h**
Blood	6.53 ± 1.33	1.25 ± 0.27	0.08 ± 0.01
Liver	3.39 ± 0.35	2.49 ± 0.31	0.75 ± 0.08
Heart	2.11 ± 0.86	0.47 ± 0.09	0.13 ± 0.01
Kidneys	5.78 ± 0.48	3.59 ± 0.60	0.88 ± 0.17
Stomach	1.09 ± 0.22	0.37 ± 0.08	0.18 ± 0.02
Intestines	2.33 ± 0.44	2.84 ± 0.38	0.32 ± 0.13
Spleen	1.63 ± 0.16	0.78±0.02	0.51 ± 0.12
Muscle	0.61 ± 0.07	0.19±0.07	0.05 ± 0.02
Lungs	3.63 ± 0.69	1.19±0.21	0.31 ± 0.16
Femur	0.97 ± 0.12	0.29 ± 0.16	0.18 ± 0.04
Pancreas	2.26 ± 0.18	0.51 ± 0.09	0.22 ± 0.01
CCK$_{2i4sv}$R(+) Tumor	12.62 ± 1.76	9.55 ± 1.344.59	2.47 ± 0.41
CCK$_{2i4sv}$R(-) Tumor	1.62 ± 0.12	0.75 ± 0.19	0.00 ± 0.00

Table S4. Biodistribution of [^{67}Ga]Ga-GAS2 in male SCID mice bearing twin HEK293-CCK$_{2i4sv}$R and wtHEK293 tumors at 1, 4 and 24 h pi, expressed as %IA/g and representing mean values ± SD, n=4.

**Organs**	**%IA/g ± SD, n=4**		
	**1 h**	**4 h**	**24 h**
Blood	0.86 ± 0.08	0.19 ± 0.06	0.05 ± 0.00
Liver	5.51 ± 2.03	2.64 ± 0.85	0.78 ± 0.17
Heart	0.27 ± 0.03	0.10 ± 0.03	0.04 ± 0.01
Kidneys	1.48 ± 0.18	0.39 ± 0.11	0.06 ± 0.01
Stomach	0.32 ± 0.14	0.14 ± 0.06	0.06 ± 0.02
Intestines	25.26 ± 2.90	26.38 ± 11.89	0.31 ± 0.32
Spleen	0.52 ± 0.06	0.55 ± 0.15	0.35 ± 0.02
Muscle	0.12 ± 0.02	0.04 ± 0.01	0.02 ± 0.00
Lungs	0.80 ± 0.09	0.34 ± 0. 01	0.15 ± 0.02
Femur	0.24 ± 0.03	0.13 ± 0.03	0.10 ± 0.03
Pancreas	0.37 ± 0.05	0.08 ± 0.05	0.03 ± 0.00
CCK$_{2i4sv}$R(+) Tumor	4.06 ± 0.07	1.53 ± 0.26	0.24 ± 0.03
CCK$_{2i4sv}$R(-) Tumor	0.48 ± 0.03	0.19 ± 0.08	0.08 ± 0.00

Table S5. Biodistribution of [^{67}Ga]Ga-GAS3 in male SCID mice bearing twin HEK293-CCK$_{2i4sv}$R and wtHEK293 tumors at 1, 4 and 24 h pi, expressed as %IA/g and representing mean values ± SD, n=4.

**Organs**	**%IA/g ± SD, n=4**		
	**1 h**	**4 h**	**24 h**
Blood	8.61 ± 1.17	2.23 ± 0.39	0.14 ± 0.02
Liver	5.62 ± 0.40	4.97 ± 0.74	2.48 ± 0.34
Heart	2.23 ± 0.38	0.76 ± 0.07	0.17 ± 0.02
Kidneys	9.09 ± 1.51	7.86 ± 0.48	1.98 ± 0.45
Stomach	1.51 ± 0.13	0.61 ± 0.12	0.19 ± 0.03
Intestines	1.53 ± 0.19	1.59 ± 0.19	0.39 ± 0.06
Spleen	1.96 ± 0.13	1.60 ± 0.39	1.67 ± 0.37
Muscle	0.82 ± 0.14	0.25 ± 0.05	0.06± 0.00
Lungs	4.32 ± 0.58	1.51 ± 0. 22	2.43 ± 1.04
Femur	1.60 ± 0.31	0.78 ± 0.10	0.52 ± 0.07
Pancreas	2.51 ± 0.22	0.77 ± 0.11	0.17 ± 0.03
CCK$_{2i4sv}$R(+) Tumor	21.75 ± 4.90	20.39 ± 3.96	5.37 ± 1.00
CCK$_{2i4sv}$R(-) Tumor	2.52 ± 0.28	1.15 ± 0.18	0.61 ± 0.08

Table S6. Biodistribution of [^{111}In]In-GAS1 in male SCID mice bearing twin HEK293-CCK$_{2i4sv}$R and wtHEK293 tumors at 4 and 24 h pi (control and Gelofusine-treated mice), expressed as %IA/g and representing mean values ± SD, n=4.

Organs	%IA/g ± SD, n=4			
	**4 h**	**4 h + Gelo**	**24 h**	**24 h + Gelo**
Blood	3.51 ± 0.56	2.75 ± 0.48	0.08 ± 0.01	0.07 ± 0.01
Liver	7.69 ± 1.00	6.34 ± 0.86	2.27 ± 0.30	1.30 ± 0.15
Heart	1.10 ± 0.19	0.92 ± 0.16	0.15 ± 0.03	0.15 ± 0.02
Kidneys	6.06 ± 1.24	5.93 ± 0.56	1.84 ± 0.38	1.69 ± 0.13
Stomach	0.98 ± 0.21	0.77 ± 0.14	0.21 ± 0.05	0.25 ± 0.04
Intestines	3.37 ± 0.89	1.87 ± 0.08	0.59 ± 0.11	0.49 ± 0.13
Spleen	1.56 ± 0.22	1.13 ± 0.28	0.64 ± 0.12	0.48 ± 0.08
Muscle	0.26 ± 0.04	0.28 ± 0.06	0.06± 0.00	0.06± 0.01
Lungs	2.23 ± 0.39	1.86 ± 0. 22	0.36 ± 0.05	0.39 ± 0.03
Femur	0.77 ± 0.13	0.65 ± 0.15	0.16 ± 0.03	0.16 ± 0.01
Pancreas	1.49 ± 0.19	1.47 ± 0.26	0.18 ± 0.03	0.14 ± 0.01
CCK$_{2i4sv}$R(+) Tumor	17.79 ± 1.73	19.17 ± 2.06	5.40 ± 1.13	4.51 ± 0.55
CCK$_{2i4sv}$R(-) Tumor	0.73 ± 0.05	0.86 ± 0.15	0.27 ± 0.10	0.23 ± 0.04

Table S7. Biodistribution of [^{111}In]In-GAS2 in male SCID mice bearing twin HEK293-CCK$_{2i4sv}$R and wtHEK293 tumors at 4 and 24 h pi (control and Gelofusine-treated mice), expressed as %IA/g and representing mean values ± SD, n=4.

Organs	%IA/g ± SD, n=4			
	**4 h**	**4 h + Gelo**	**24 h**	**24 h + Gelo**
Blood	6.40 ± 0.75	6.29 ± 1.08	0.13 ± 0.02	0.15 ± 0.02
Liver	5.42 ± 0.89	4.56 ± 0.49	0.96 ± 0.22	0.95 ± 0.12
Heart	1.61 ± 0.23	1.42 ± 0.21	0.22 ± 0.01	0.22 ± 0.03
Kidneys	29.55 ± 3.64	21.90 ± 3.97	10.16 ± 2.30	11.51 ± 2.64
Stomach	1.31 ± 0.21	0.94 ± 0.15	0.36 ± 0.14	0.35 ± 0.06
Intestines	9.71 ± 0.42	9.48 ± 0.99	0.68 ± 0.15	0.75 ± 0.06
Spleen	1.49 ± 0.14	1.32 ± 0.20	0.51 ± 0.09	0.56 ± 0.17
Muscle	0.56 ± 0.08	0.47 ± 0.04	0.08 ± 0.01	0.08± 0.01
Lungs	3.27 ± 0.46	2.72 ± 0. 30	0.60 ± 0.37	0.44 ± 0.07
Femur	1.15 ± 0.10	0.99 ± 0.17	0.24 ± 0.04	0.25 ± 0.03
Pancreas	2.99 ± 0.96	3.00 ± 0.58	0.29 ± 0.03	0.30 ± 0.05
CCK$_{2i4sv}$R(+) Tumor	8.91 ± 1.59	7.67 ± 0.61	2.78 ± 0.43	2.92 ± 0.37
CCK$_{2i4sv}$R(-) Tumor	1.82 ± 0.25	1.72 ± 0.30	0.41 ± 0.10	0.45 ± 0.09

Table S8. Biodistribution of [^{111}In]In-GAS3 in male SCID mice bearing twin HEK293-CCK$_{2i4sv}$R and wtHEK293 tumors at 4 and 24 h pi, expressed as %IA/g and representing mean values ± SD, n=4.

Organs	%IA/g ± SD, n=4	
	**4 h**	**24 h**
Blood	2.42 ± 0.94	0.08 ± 0.01
Liver	2.78 ± 0.27	1.07 ± 0.26
Heart	0.81 ± 0.28	0.16 ± 0.02
Kidneys	9.32 ± 0.68	4.15 ± 1.06
Stomach	0.91 ± 0.39	0.35 ± 0.21
Intestines	1.66 ± 0.33	0.48 ± 0.14
Spleen	0.69 ± 0.12	0.43 ± 0.09
Muscle	0.31 ± 0.13	0.08 ± 0.01
Lungs	1.36 ± 0.37	0.34 ± 0. 07
Femur	0.60 ± 0.14	0.27 ± 0.09
Pancreas	0.86 ± 0.23	0.17 ± 0.04
CCK$_{2i4sv}$R(+) Tumor	19.83 ± 1.35	11.53 ± 1.93
CCK$_{2i4sv}$R(-) Tumor	0.85 ± 0.37	0.40 ± 0.21

Table S9. Biodistribution of [^{177}Lu]Lu-GAS1 in male SCID mice bearing twin HEK293-$CCK_{2i4sv}R$ and wtHEK293 tumors at 4, 24, 48, 72 and 96 h pi, expressed as %IA/g and representing mean values ± SD, n=4.

Organs	%IA/g ± SD, n=4				
	**4 h**	**24 h**	**48 h**	**72 h**	**96 h**
Blood	0.99 ± 0.20	0.06 ± 0.00	0.04 ± 0.01	0.04 ± 0.00	0.04 ± 0.00
Liver	2.21 ± 0.44	0.51 ± 0.07	0.32 ± 0.04	0.23 ± 0.02	0.19 ± 0.02
Heart	0.41 ± 0.07	0.12 ± 0.00	0.07 ± 0.01	0.05 ± 0.01	0.04 ± 0.00
Kidneys	3.70 ± 0.62	1.05 ± 0.12	0.38 ± 0.07	0.14 ± 0.04	0.10 ± 0.04
Stomach	0.37 ± 0.05	0.14 ± 0.03	0.08 ± 0.01	0.05 ± 0.01	0.05 ± 0.01
Intestines	1.65 ± 0.28	0.32 ± 0.07	0.08 ± 0.01	0.06 ± 0.02	0.08 ± 0.05
Spleen	0.59 ± 0.07	0.32 ± 0.01	0.26 ± 0.06	0.51 ± 0.71	0.14 ± 0.02
Muscle	0.12 ± 0.02	0.04 ± 0.00	0.03 ± 0.00	0.02 ± 0.00	0.02 ± 0.01
Lungs	0.94 ± 0.15	0.34 ± 0.03	0.29 ± 0.04	0.21 ± 0.03	0.17 ± 0.02
Femur	0.28 ± 0.03	0.11 ± 0.01	0.09 ± 0.01	0.06 ± 0.00	0.05 ± 0.01
Pancreas	0.56 ± 0.11	0.10 ± 0.01	0.05 ± 0.01	0.03 ± 0.00	0.03 ± 0.01
$CCK_{2i4sv}R$(+) Tumor	8.74 ± 1.60	2.09 ± 0.50	0.97 ± 0.20	0.35 ± 0.23	0.56 ± 0.25
$CCK_{2i4sv}R$(-) Tumor	1.01 ± 0.30	0.29 ± 0.20	0.17 ± 0.02	0.18 ± 0.05	0.11 ± 0.04

Table S10. Biodistribution of [^{177}Lu]Lu-GAS3 in male SCID mice bearing twin HEK293-$CCK_{2i4sv}R$ and wtHEK293 tumors at 1, 4, 24, 48, 72 and 96 h pi, expressed as %IA/g and representing mean values ± SD, n=4.

Organs	%IA/g ± SD, n=4				
	**4 h**	**24 h**	**48 h**	**72 h**	**96 h**
Blood	3.19 ± 0.44	0.07 ± 0.01	0.05 ± 0.01	0.04 ± 0.00	0.04 ± 0.00
Liver	2.73 ± 0.31	0.51 ± 0.05	0.35 ± 0.09	0.32 ± 0.02	0.25 ± 0.02
Heart	0.94 ± 0.21	0.08 ± 0.02	0.08 ± 0.01	0.04 ± 0.01	0.03 ± 0.01
Kidneys	8.86 ± 0.67	1.42 ± 0.29	0.47 ± 0.07	0.17 ± 0.04	0.11 ± 0.03
Stomach	0.64 ± 0.07	0.11 ± 0.01	0.08 ± 0.01	0.06 ± 0.02	0.04 ± 0.00
Intestines	1.85 ± 0.18	0.17 ± 0.04	0.07 ± 0.01	0.04 ± 0.01	0.03 ± 0.01
Spleen	1.19 ± 0.12	0.45 ± 0.05	0.43 ± 0.10	0.44 ± 0.07	0.39 ± 0.02
Muscle	0.31 ± 0.04	0.03 ± 0.01	0.04 ± 0.01	0.03 ± 0.01	0.01 ± 0.00
Lungs	1.94 ± 0.21	0.26 ± 0.02	0.18 ± 0.03	0.15 ± 0.01	0.14 ± 0.01
Femur	0.60 ± 0.07	0.11 ± 0.02	0.14 ± 0.04	0.06 ± 0.00	0.06 ± 0.00
Pancreas	1.00 ± 0.13	0.07 ± 0.01	0.08 ± 0.04	0.03 ± 0.01	0.02 ± 0.00
$CCK_{2i4sv}R$(+) Tumor	20.88 ± 1.20	5.38 ± 0.77	3.39 ± 0.23	1.99 ± 0.31	1.52 ± 0.43
$CCK_{2i4sv}R$(-) Tumor	1.14 ± 0.50	0.37 ± 0.12	0.25 ± 0.14	0.13 ± 0.03	0.00 ± 0.04

References

Milliken, B.T.; Doyle, R.P.; Holz, G.G.; Chepurny, O.G. FRET reporter assays for cAMP and calcium in a 96-well format using genetically encoded biosensors expressed in living cells. *Bio Protoc*. **2020**, *10* (11), e3641.

Chepurny, O.G.; Matsoukas, M.T.; Liapakis, G.; Leech, C.A.; Milliken, B.T.; Doyle, R.P.; Holz, G.G. Nonconventional glucagon and GLP-1 receptor agonist and antagonist interplay at the GLP-1 receptor revealed in high-throughput FRET assays for cAMP. *J. Biol. Chem*. **2019**, *294* (10), 3514-3531. Erratum in: *J. Biol. Chem*. **2019**, *294* (22), 8714.
